# Immunogenicity and efficacy of mRNA COVID-19 vaccine MRT5500 in preclinical animal models

**DOI:** 10.1038/s41541-021-00324-5

**Published:** 2021-04-19

**Authors:** Kirill V. Kalnin, Timothy Plitnik, Michael Kishko, Jinrong Zhang, Donghui Zhang, Adrien Beauvais, Natalie G. Anosova, Tim Tibbitts, Josh DiNapoli, Gregory Ulinski, Peter Piepenhagen, Sheila M. Cummings, Dinesh S. Bangari, Susan Ryan, Po-Wei D. Huang, James Huleatt, Deanne Vincent, Katherine Fries, Shrirang Karve, Rebecca Goldman, Hardip Gopani, Anusha Dias, Khang Tran, Minnie Zacharia, Xiaobo Gu, Lianne Boeglin, Jonathan Abysalh, Jorel Vargas, Angela Beaulieu, Monic Shah, Travis Jeannotte, Kimberly Gillis, Sudha Chivukula, Ron Swearingen, Victoria Landolfi, Tong-Ming Fu, Frank DeRosa, Danilo Casimiro

**Affiliations:** 1 Sanofi Pasteur, Cambridge, MA USA; 2 Yoh Services LLC, Cambridge, MA USA; 3 Sanofi Pasteur, Swiftwater, PA USA; 4 Global Discovery Pathology, Sanofi, Framingham, MA USA; 5 Translate Bio, Lexington, MA USA; 6 Sanofi Pasteur, Marcy l’Etoile, France

**Keywords:** Biotechnology, Immunology, Microbiology, Diseases, Pathogenesis

## Abstract

Emergency use authorization of COVID vaccines has brought hope to mitigate pandemic of coronavirus disease 2019 (COVID-19). However, there remains a need for additional effective vaccines to meet the global demand and address the potential new viral variants. mRNA technologies offer an expeditious path alternative to traditional vaccine approaches. Here we describe the efforts to utilize an mRNA platform for rational design and evaluations of mRNA vaccine candidates based on the spike (S) glycoprotein of SARS-CoV-2. Several mRNA constructs of S-protein, including wild type, a pre-fusion stabilized mutant (2P), a furin cleavage-site mutant (GSAS) and a double mutant form (2P/GSAS), as well as others, were tested in animal models for their capacity to elicit neutralizing antibodies (nAbs). The lead 2P/GSAS candidate was further assessed in dose-ranging studies in mice and *Cynomolgus* macaques, and for efficacy in a Syrian golden hamster model. The selected 2P/GSAS vaccine formulation, designated MRT5500, elicited potent nAbs as measured in neutralization assays in all three preclinical models and more importantly, protected against SARS-CoV-2-induced weight loss and lung pathology in hamsters. In addition, MRT5500 elicited T_H_1-biased responses in both mouse and non-human primate (NHP), thus alleviating a hypothetical concern of potential vaccine-associated enhanced respiratory diseases known associated with T_H_2-biased responses. These data position MRT5500 as a viable vaccine candidate for entering clinical development.

## Introduction

SARS-CoV-2, previously known as the 2019-novel coronavirus (2019-nCoV)^1^, is a β-coronavirus with a yet-to-be defined zoonotic origin. The first cases of human infection with severe acute respiratory syndrome (SARS) were reported in December 2019 in China^2^, and the condition was later named coronavirus disease 2019 (COVID-19)^3^. In contrast to SARS-CoV-1 virus which caused an outbreak in 2002, SARS-CoV-2 has gained high capacity for human-to-human transmission and quickly spread worldwide. It has caused over 100 million cases of confirmed infection and more than 2,400,000 deaths (https://www.worldometers.info/coronavirus). Widespread efforts have been made towards the rapid development of vaccines with several effective vaccines recently approved for emergency use^4,5^. While the success and speed of development has been remarkable, there remains a demand for continued vaccine development for addressing global public health mandate.

Coronavirus is an enveloped RNA virus, and the viral spike (S) protein on the virion envelope is essential for infection and the target for host antiviral antibodies^6,7^. The receptor for SARS-CoV-2 is angiotensin-converting enzyme 2 (ACE2), a metalloprotease that also serves as the receptor for SARS-CoV-1^8^. Most of the COVID-19 vaccine candidates reported are focused on a pre-fusion-stabilized S protein, either expressed as recombinant protein or delivered from viral vectors or as DNA or mRNA vaccines^9–17^. The pre-fusion-stabilized version of SARS-CoV-2 S-protein contains two proline substitutions (2P), at amino acid positions 986 and 987, located near the apex of the central helix and heptad repeat 1^18^. Structural studies reveal that the pre-fusion stabilized S closely resembles native S protein on the virion surface; a structure targeted by many reported effective neutralizing antibodies^19–21^. Moreover, the vaccine premises are based on the prior work of MERS-CoV, SARS-CoV and HCoV-HKU1 S proteins presented in pre-fusion conformations^22–24^. The ability of S-2P-based vaccines to elicit neutralizing antibodies has been demonstrated^9–11,25,26^.

There is a unique feature of SARS-CoV-2 S protein which possesses a polybasic furin cleavage site at the junction of S1 and S2 subunits. This feature is believed to have emerged during viral transmission from its zoonotic host to human^27–29^, and is one of the key attributes to the high transmissibility of SARS-CoV-2 in humans^30,31^. Interestingly, both cleaved and uncleaved versions of S protein co-exist on virions purified from viral culture on Vero cells^32,33^. Thus, it remains unclear how the cleavage provides an advantage for viral transmission. Also, from a vaccine design perspective, one can speculate that furin cleavage may lead to subtle conformational changes in the trimerized S protein, potentially favoring its interaction with ACE2^28,34^.

These unanswered questions led us to design mRNA constructs encoding various forms of S constructs to evaluate effects of the cleavage site and various prefusion mutations on vaccine immunogenicity. The stabilization could be achieved through double proline mutations^7^ and the furin cleavage can be abrogated by cleavage site mutations; these mutations are herein referred to as 2P and GSAS mutations, respectively. In addition, we eliminated an endoplasmic reticulum (ER) retention signal located at the C-terminus which has been reported to be beneficial for cell surface expression of the SARS-CoV-1 S protein^34^. Lastly, a recent report indicated that four proline mutations in addition to 2P, at positions of 817, 892, 899 and 942, could further stabilize the prefusion form, thus prompted us to add these constructs for evaluation^35^. These constructs were examined for expression and cellular trafficking in vitro and immunogenicity in mice or non-human primates (NHPs). The 2P/GSAS S mRNA encapsulated in an ionizable lipid nanoparticle (LNP) formulation, designated MRT5500, was subsequently selected for further evaluation for protection efficacy in Syrian golden hamsters. Our studies demonstrated the ability of MRT5500 to induce both humoral and cell-mediated antiviral responses and confer protection against a high virulent challenge in hamsters, thus supporting its further development as a clinical candidate.

## Results

### Design and in vitro evaluation of mRNA constructs

SARS-CoV-2 S protein, a 1273 amino acid glycoprotein, is expressed as a membrane anchored homo-trimer^7^. The receptor binding domain (RBD) has been identified as the critical component to initiate virus attachment to ACE2, a cellular receptor for viral infection^36^. Interestingly, the RBD is present in both up and down configurations in the pre-fusion form of S protein, and the up position has been speculated as the prerequisite for interaction with ACE2^7,32^. The furin cleavage at the S1/S2 boundary of SARS-CoV-2 S occurs during viral biosynthesis^37^. It is postulated that transition and adaptation to the human host resulted in the acquisition of a furin protease site in the S protein of SARS-CoV-2, a unique feature not found in SARS-CoV-1 and other SARS-related-CoVs^28^. Approximately 45% of the total S protein monomers presented within intact SARS-CoV-2 virions have been reported as cleaved at the furin cleavage site;^32^ however, it is not clear which form is favored by the virus to facilitate the fusion process^28,37–39^.

**Fig. 1:**
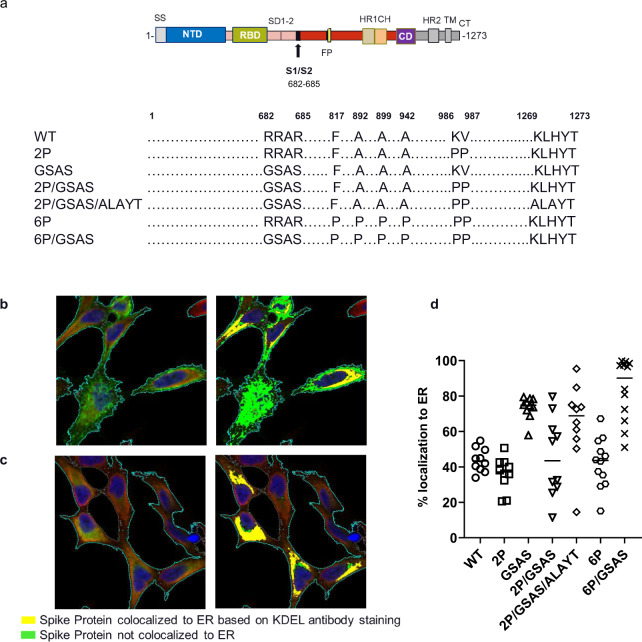
Intracellular localization of S antigen constructs expressed in HeLa cells. Schemata of full-length S-genes used in vaccine selection study (**a**). Intracellular localization of S antigens expressed in HeLa cells after transient transfection of mRNA constructs. Spike Protein expression (green) of 2 P mutant (**b**) and GSAS mutant (**c**) is analyzed along with KDEL an endoplasmic reticulum marker (red). Left panels represent transmitted light microscope image with visualized S-signal, while right panels represent overlay of Spike/KDEL. Quantitative representation of colocalization of S-protein signal with ER marker per mRNA variant based on fluorescence signals (**d**, see also Supplementary Figs. 1–8).

The COVID-19 vaccine hypothesis has been centered around induction of neutralizing antibodies (nAbs) that either block the interaction of the RBD with ACE2, or prevent the fusion process involving S protein transition from pre- to post-fusion conformation^40,41^. Although the pre-fusion conformation is known to be critical for eliciting a neutralizing response^20,21^, the impact of the furin cleavage site in eliciting neutralizing antibodies is unclear. Thus, we mutated the furin cleavage site of RRAR to GSAS at position 682 to 685^38,42^. To maximize stabilizing S and to understand different mutations on its expression and immunogenicity, we synthesized seven codon-optimized mRNA constructs based on the full-length SARS-CoV-2 S protein, to represent wild type (WT), a stabilized pre-fusion mutant (2P)^20^, a furin cleavage site mutant (GSAS), a double mutant (2P/GSAS), a triple mutant incorporating 2P/GSAS mutations with the mutation of a C-terminal ER retention signal (KLHYT)^34^, and a six proline replacements variant (6P)^18^ with or without GSAS mutations (Fig. 1a).

To understand the effects of these mutations on the S protein expression and subcellular location, we transfected HeLa cells (HeLa) and visualized for S-protein by immunostaining and confocal microscopy. Cells were permeabilized and stained with antibodies for S-protein and KDEL, an endoplasmic reticulum (ER) marker^43,44^ (Fig. 1b, c, Supplementary Figs. 1–7). ER, with extension to ER–Golgi intermediate compartment (ERGIC)^34,45^, is the translocation site for coronavirus structural proteins to assemble and encapsulate progeny viral genome to form mature virions^46^. All GSAS mutants revealed strong signals for colocalization with the ER marker, while WT, 2P or 6P variants appeared to be expressed and presented on the cell surface (Fig. 1d, Supplementary Fig. 8). We calculated the percent of cells with S-protein expression colocalized or not colocalized within the ER for each mRNA variant using 10 randomly selected image fields per mRNA variant (Supplementary Figs. 1–7). Interestingly, 2P/GSAS and 2P/GSAS/ALAYT constructs revealed a mixed phenotype with both ER and cell surface expression. We estimated cell surface expression using visual observations (Supplementary Fig. 8). Thus, our data demonstrated that the different mutations could impact the S-protein trafficking pattern through secretory pathways and affect its rate of delivery to be presented on the cell surface. Understanding the mechanisms by which these mutations lead to their impact on ER retention would require further investigation.

### Selection of lead mRNA constructs

To determine the impact of these mutations on immunogenicity, we formulated each of the seven mRNA constructs within a LNP as mRNA vaccines drug products^47^ and assessed their respective immune responses in two animal models. BALB/c mice were administered two immunizations at a three-week interval with a 0.4 µg per dose of each of five formulations (WT, 2P, GSAS, 2P/GSAS, 2P/GSAS/ALAYT). In parallel, NHPs were immunized using the same immunization schedule at 5 µg per dose of six S mRNA vaccines (2P, GSAS, 2P/GSAS, 2P/GSAS/ALAYT, 6P and 6P/GSAS).

**Fig. 2:**
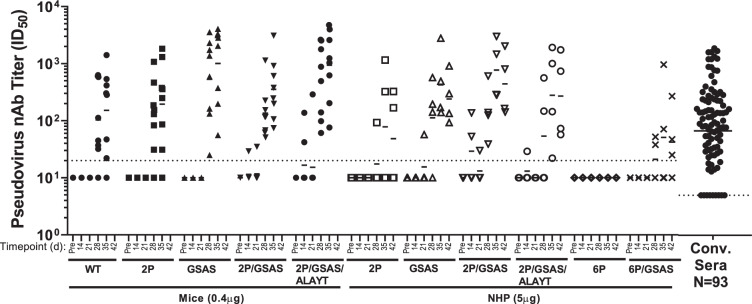
2P/GSAS Spike modification constructs evaluated in mice and NHPs for neutralization potency. Serum neutralizing titers in mice and NHPs immunized with mRNA vaccines. BALB/c female mice (*n* = 8) or Cynomolgus monkeys (*n* = 4) were immunized twice three weeks apart with 0.4 or 5 µg of mRNA vaccines formulations respectively. Serum samples collected at indicated dates were tested in a pseudovirus neutralization assay (PsVNa). The 50% inhibitory dilution titers (ID_50_) were calculated as the reciprocal of the dilution that reduced the number of virus entry events by 50%. Each dot represents an individual serum sample, and the line represents the geometric mean for the group. The dotted line in each panel represents the lower limit of assay readout.

To evaluate nAbs titers, we tested the ability of immune sera to neutralize the infectivity of GFP reporter pseudoviral particles (RVP) in HEK-293T cells stably over-expressing human ACE2^48^ (Fig. 2). RVPs expressing SARS CoV-2 S protein are capable of a single round of infection, indicated by GFP expression upon entry. Neutralizing potency was determined as the serum dilution which can achieve 50% inhibition of RVP entry (ID_50_). In addition, we evaluated ELISA titers using either a recombinant S-protein monomer or a recombinant soluble S-protein trimerized by GCN4 helix bundle as an antigen^49^ (Supplementary Fig. 9b, c).

Although a few mice developed neutralizing titers at D14 after the first immunization, the titers were in general low. Expectedly, majority of animals developed neutralizing titers after the second immunization (Fig. 2). On D35, the geometric mean titers (GMTs) with the 95% confidence interval (95% CI) for pseudoviral (PsV) nAb titers in mice were 152 (36; 645) for WT, 195 (44; 870) for 2P, 1005 (261; 3877) for GSAS, 354 (129; 976) for 2P/GSAS and 940 (280; 3154) for 2P/GSAS/ALAYT. It was noted that there was a high variability within each experimental group, and the GMTs with the corresponding CI were overlapping for most of the mRNA constructs, indicating that it would be difficult to claim any statistical difference when ranking these mRNA vaccines. However, there was a trend for higher GMTs, especially at D35 and D42, for three constructs with GSAS mutations when compared to those of WT and 2P constructs.

In NHPs, we also observed diverse neutralizing titers within each group even after the second immunization (Fig. 2). 2P and 6P/GSAS vaccines showed lower immunogenicity than other constructs with GMTs of 78 and 51 at D35, respectively. Surprisingly, 6P vaccine failed to elicit any detectable neutralizing titers. Consistent with the observations in the mouse study, all GSAS constructs with the exception of 6P/GSAS induced higher neutralizing titers after the second dose, with GMTs (95% CI) at D35 recorded as 425 (48; 3769) for GSAS, 772 (116; 5121) for 2P/GSAS, 280 (11; 6970) for 2 P/GSAS/ALAYT, as compared to those of comprising just the 2P mutation vaccine group. Although the study was not designed with sufficient power for statistical differentiation, the trending of GMTs in both mice and NHPs suggested superior immunogenicity for 2P/GSAS to other constructs (Supplementary Table 9). We also demonstrated that the peak PsVNa titers (D35) for the 2P/GSAS variant in mice and NHPs were higher than the titers observed in a panel of 93 convalescent sera from COVID-19 patients.

Another important observation is that ELISA titers were not always predictive of neutralizing titers by PsVNa (Fig. 2 and Supplementary Fig. 9b, c). In mice, all five vaccines demonstrated similar levels of binding antibodies 14 days after the first immunization, and the responses were further enhanced one week after the second dose at day (D) 28. On D35, the IgG GMTs for WT, 2P, GSAS and 2P/GSAS groups were 184,343, 200,896, 379,653 and 201,080 respectively. There were no statistically significant differences among those GMT titers. In contrast, in NHPs ELISA titers were well correlated with PsVNa titers in both magnitude and dynamic of response. On D35 NHP binding IgG GMTs were recorded as 6459, 132,181, 76,315, 16,849, 714, and 5616 for 2P, GSA, 2P/GSAS, 2P/GSAS/ALAYT, 6P, 6P/GSAS respectively.

With these evaluations, we determined that the GSAS mutation was beneficial for vaccine immunogenicity. The 2P mutation, which was introduced for stabilization of prefusion form of S protein, appeared beneficial in the context of the GSAS mutation, while ALAYT showed less impact on immunogenicity, especially in NHPs, in the context of 2P/GSAS. Thus, we selected the double mutant 2P/GSAS in a novel LNP formulation, designated MRT5500, for further preclinical evaluations.

### Serological evaluations of MRT5500 in mice and NHPs

**Fig. 3:**
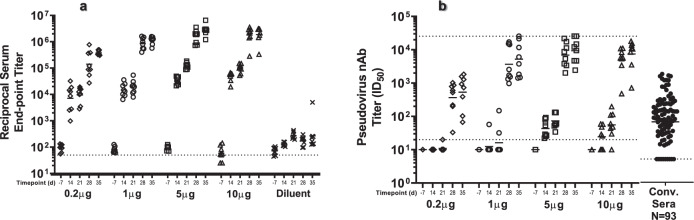
Serological evaluation of MRT5500 formulation in mice. Groups of BALB/c mice (*n* = 8) were immunized at D0 and D21 with 0.2, 1.0, 5.0 or 10.0 µg dose of MRT5500 formulation. Serum samples at the indicated time were tested in ELISA (**a**) and PsVNa (**b**). Each symbol represents a serum sample and the line is the geometric mean of the group. The dotted lower line in each panel represents the lower limit of assay detection, while dotted upper line in panel **b** represents the upper limit of assay detection. PsV neutralization titers (nAb) of the human convalescent serum panel (*n* = 93) were defined in separate experiment and shown in the same scale on *Y*-axis as other samples.

MRT5500 was evaluated for immunogenicity in a range of doses relevant to the projected human dose levels. Four dose levels in mice were assessed, ranging from 0.2 to 10 µg per dose. As expected, MRT5500 induced dose-dependent S-specific binding antibodies and neutralizing antibodies in mice (Fig. 3a). PsVNa titers were detected in the higher dose groups (5, 10 µg) after the first immunization, with the titers being more pronounced after the second immunization at D21. The PsVNa GMTs were 534, 5232, 9370 and 7472 at D35 for the 0.2, 1.0, 5.0 and 10.0 μg dose groups, respectively. However, there were no statistically significant differences in PsV neutralization titers at D35 between 1, 5 and 10 µg groups (Supplementary Table 1), which may indicate a dose-saturation effect beyond 1 µg in mice. We also demonstrated that the peak PsV titers (D35) in mice were significantly higher than those observed in a panel of 93 convalescent sera from COVID-19 patients (Supplementary Fig. 13).

**Fig. 4:**
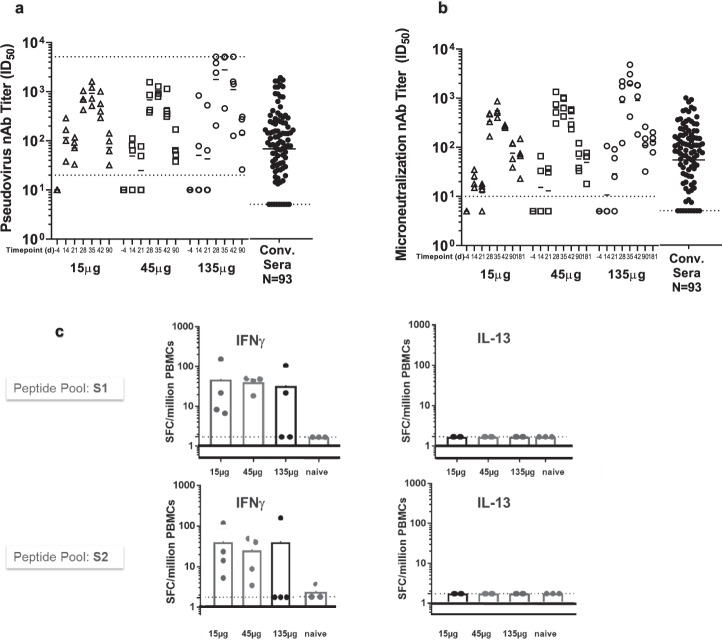
Neutralizing titers and T-cell response in NHPs vaccinated with MRT5500 formulation. Groups of cynomolgus macaques (*n* = 4) were vaccinated with MRT5500 at 15, 45 or 135 μg per dose at D0 and D21, and serum samples collected at the indicated timepoints were tested in PsVNa (**a**) and MN assay (**b**). Each symbol represents an individual sample and the line geometric means for the group. The neutralization titer of the sample, shown as ID_50_, was defined as the reciprocal of the highest test serum dilution for which the virus infectivity was reduced by 50%. PsV and MN neutralization titers (NAb) of the human convalescent serum panel (*n* = 93) were defined in separate experiment and shown in the same scale on *Y*-axis as other samples. T-cell responses in NHPs vaccinated with MRT5500 were assessed with ELISPOT assay (**c**). PBMCs collected at D42 were incubated with two peptide pools to represent the entire S open reading frame. The responses of PBMC secreting IFN-γ (left panels) or IL-13 (right panels) were calculated as spot forming cells (SFC) per million PBMC. Each symbol represents an individual sample, and the bar represent the geometric mean for the group. The dotted lower line in each panel represents the lower limit of assay detection, while dotted upper line in panel 4a represents the upper limit of assay detection.

In NHPs, we evaluated 15, 45 or 135 µg per dose (Fig. 4). After the first immunization, 10 out 12 NHPs developed antibodies reactive to recombinant S protein in ELISA, and the titers were further enhanced after a second immunization at D35 (Supplementary Fig. 11). The neutralization potency was assessed by two methods: PsVNa and microneutralization (MN) assay. In both assays, a dose-dependent increase in neutralization titer was observed, with GMTs on D35 of 924 for 15 µg, 961 for 45 µg, and 2781 for 135 µg in PsVNa (Fig. 4a). The MN GMTs followed a similar trend, with titers of 555 for 15 µg, 719 for 45 µg and 1877 for the135 µg group (Fig. 4b, Supplementary Fig. 12). Despite the observed trend towards higher titers with increasing dose, the differences between groups were not statistically significant for either MN or PsV neutralization titers.

The results from both assays were highly correlated (Supplementary Table 2). Regardless of the dose level tested, D35 PsV and MN titers were approximately 130-fold higher than those of pre-immune animals. Furthermore, the observed PsV and MN titers were significantly higher as compared to titers observed in a panel of 93 convalescent sera from COVID-19 patients (Supplementary Fig. 14).

### T-cell profiles of MRT5500 in multiple species

Vaccine associated enhanced respiratory disease (VAERD) has been a safety concern for COVID-19 vaccines in development, although the concern at this stage is only a theoretical one^50^. This phenomenon has been reported for whole-inactivated virus vaccines against measles and respiratory syncytial virus (RSV), which were tested in the 1960s (citations in ref. ^50^), and one of the disease hypotheses implicates T_H_2-biased antigen-specific CD4 T cells marked by production of IL-4, IL-5, or IL-13. A similar association between a T_H_2 profile and disease enhancement has been reported for an inactivated SARS-CoV-1 vaccine in mice^51^. Furthermore, less severe cases of SARS were associated with accelerated induction of T_H_1 cell responses^52^, whereas T_H_2-biased responses have been associated with enhancement of lung disease following infection in mice parenterally vaccinated with inactivated SARS-CoV viral vaccines^51,53^. Similar phenomena have been observed in humans. For example, a SARS-CoV-2-specific cellular response was associated with severity of disease: recovered patients with mild COVID-19 illnesses demonstrated high levels of IFN-γ induced by SARS-Cov-2 antigens, while severe pneumonia patients showed significantly lower level of this cytokine^54^. Thus, it is important to understand the T cell profiles induced by MRT5500.

T cell cytokine responses were tested in NHPs three weeks after the second immunization. Cytokines induced by re-stimulation with the pooled SARS CoV-2 S protein peptides were assessed in PBMCs on D42 by the IFN-γ (T_H_1 cytokine) and IL-13 (T_H_2 cytokine) ELISPOT assays. Majority of animals in the three dose level groups tested (10 out of 12) demonstrated the presence of IFN-γ secreting cells, ranging from five to over 100 spot-forming cells per million PBMCs. A dose-response was not observed as the animals in the lower and higher dose level groups showed comparable frequencies of IFN-γ secreting cells. In contrast, the presence of IL-13 cytokine secreting cells was not detected in any of the groups tested and at any dose level, suggesting induction of a T_H_1-biased cellular response (Fig. 4c).

A similar assessment of cellular immune responses was performed in immune splenocytes in BALB/c mice at D35. ELISPOT was conducted in the 5 and 10 µg dose groups (Supplementary Fig. 10). Even though BALB/c mice have a strong tendency for T_H_2 biased immune responses, the splenocytes from the MRT5500 immunized mice secreted predominantly IFN-γ while IL-5 responses were marginal, suggesting a considerable T_H_1 bias. Thus, the data, although with limited sets of cytokines, suggested that MRT5500 immunization elicited predominantly T_H_1-biased responses in both animal species.

### Protective efficacy of MRT5500 in Syrian golden hamsters

SARS-CoV-2 infection in hamster is a newly developed pathology model^55–57^, where the viral infection is associated with high levels of virus replication with peak titers in the lungs and nasal epithelia at 2 day post-infection (DPI), histopathological evidence of disease in lungs at 7 DPI^58^, and about 8–15% weight loss around 7 DPI^55–57,59^.

To evaluate the potential of MRT5500 to protect against the viral infection and disease, we immunized hamsters with four vaccine formulation dose levels of 0.15, 1.5, 4.5 or 13.5 µg per dose, either as a single IM immunization at D21 or two IM administrations at D0 and D21. Animals were challenged at D49 via intranasal (IN) inoculation of SARS-CoV-2 and monitored for clinical manifestations of disease as daily body weight loss. Lungs and nasal tissues were harvested at 4 or 7 DPI for histopathology, and for quantification of viral replication by subgenomic RNA (sgRNA) RT-qPCR assays.

MRT5500 induced robust dose-dependent binding and neutralizing antibody responses after the first immunization, further enhanced by the second immunization (Supplementary Fig. 16). After the first immunization, all animals, except those in the group with 0.15 µg dose group, developed neutralizing antibodies recorded as plaque reduction neutralization titers (PRNT) against wild-type SARS-CoV-2 virus. Pre-challenge D35 PRNT_50_ GMTs for single dose immunization schedules were 237, 410 and 711 for a 1.5, 4.5 and 13.5 µg dose respectively, while corresponding values for two-dose groups were 1858, 2806 and 3693. Despite the observed trend towards higher titers with increasing dose, the differences between titers in the 1.5, 4.5 and 13.5 µg groups were not statistically significant (except for one-dose regimen, between 1.5 and 13.5 µg groups).

**Fig. 5:**
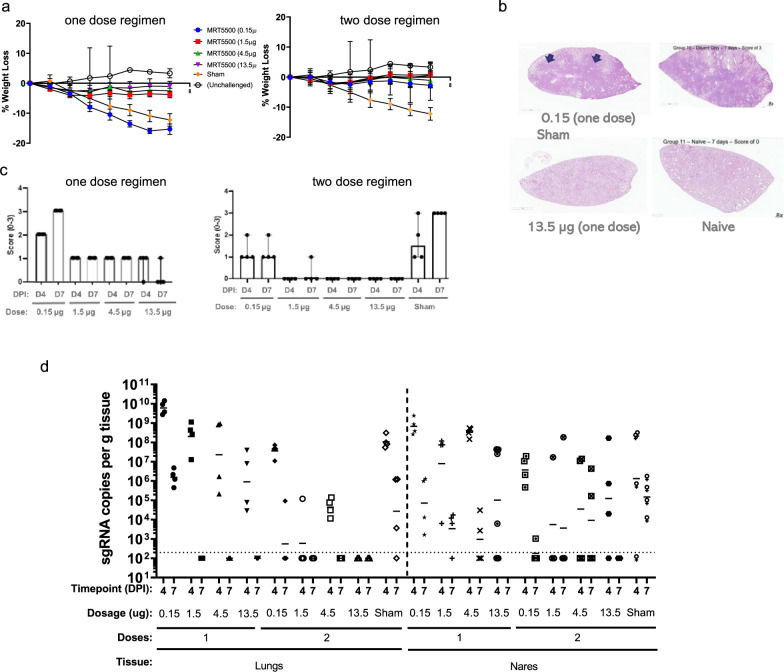
Protective efficacy of MRT5500 in a hamster model. **a** weight loss post challenge for hamsters received one (left panel) or two doses (right panel) of 0.15, 1.5, 4.5.or 13.5 µg of MRT5500 vaccine; **b** H&E staining of lungs of animals received one dose MRT5500 of either 0.15 µg or from Sham vaccinated and unchallenged (Naïve) animals. **c** D4 and D7 post-challenge (DPI) pathogenicity scores (see the “Methods” section) of animals immunized with either one (left panel)) or two (right panel) dose regimens of MRT5500; **d** Quantification of SARS-CoV-2 subgenomic RNA (sgRNA) in lungs and nares of hamsters immunized with one or two doses of MRT5500 as compared to control (Sham and Naïve) on Day 4 and Day 7 post-infection (DPI; see Supplementary Table 10).

After challenge, the body weight for each animal was monitored daily for 7 days (Fig. 5a**;** Supplementary Tables 3–8). Sham (diluent) vaccinated animals demonstrated a trend to greater degree of weight loss, with more than 10% at 7 DPI, however, due to small sample size, the definitive statistical significance could not be demonstrated. The immunization regimens of 1.5, 4.5, and 13.5 µg, regardless of one dose or two dose regimens, protected animals against body weight loss, with most animals experiencing less than 5% loss, mostly around 2–3 DPI (Supplementary Tables 3–8). Protection from weight loss was observed from immunization as low as 0.15 µg given as a two-dose regimen. The only group experiencing a similar degree of weight loss, compared to that of sham group, was the 0.15 µg dose group with single immunization.

To assess the pathology caused by viral infection, lung samples were harvested from 4 animals of each group at either 4 or 7 DPI, and the fixed tissues were sectioned, randomized, and blinded for histopathological examination. A pathology score of 0–3 was assigned to each sample based on distribution of pulmonary pathology severity of tissue damages, with the higher score reflecting the more widespread tissue involvement. A score of 1 was attributed to lung sections that revealed histopathology findings in less than 25% of the section. Similarly, if greater than 25% but <50% of the parenchyma was involved, a score of 2 was assigned. A score of 3 was designated to those sections where more than 50% of the total section was affected. Sham vaccinated hamsters inoculated with SARS-CoV-2 revealed widespread lung histopathology which resemble the reports of severe pneumonia detected COVID-19 patients (Fig. 5b, Supplementary Figs. 15, 17, 18). In these animals, lung architecture was severely disrupted multifocally or focally by marked inflammatory infiltrate composed of macrophages, lymphocytes, heterophils and syncytial cells admixed with hemorrhage and cellular debris. There was marked type 2 pneumocyte hyperplasia. Bronchiolar epithelium was frequently hyperplastic, lined by epithelial cells exhibiting piling up interspersed with rare necrotic cells. Necrotic cellular debris occupied the lumen of some affected bronchioles. Blood vessels revealed either mural or perivascular infiltration by mixed inflammatory infiltrates. Multifocally, alveolar spaces were lined by highly proliferative and pleomorphic epithelial cells. Lungs from naïve hamsters (healthy controls) were histologically unremarkable. Similar lesions could be seen in lung samples from the 0.15 μg dose group of single immunization, which was scored as 3 in blind examination. Conversely, lung samples from the single immunization 13.5 μg dose group were attributed scores of 0 as no pathology was observed similar to that of healthy controls. (Fig. 5c).

Lung pathology was markedly attenuated in hamsters that received either one or two doses of the MRT5500 vaccine, and there appeared to be a dose-dependent effect at both 4 and 7 DPI (Fig. 5c). While a single immunization of 1.5, 4.5 and 13.5 μg can attenuate pathology, the two-dose immunization of 1.5, 4.5 and 13.5 μg provided almost complete protection against pathology. The very low dose level of 0.15 μg showed no protection when used in a single-dose regimen but some marginal protection in a two-dose immunization regimen.

To assess whether MRT5500 immunization could impact viral infection in hamsters, we measured viral subgenomic RNA (sgRNA) from lung and nasal samples by RT-qPCR. Lung and nasal samples of half the group (n = 4) were collected at either 4 or 7 DPI and total RNA was processed for detection of sgRNA by RT-qPCR (Fig. 5d). For lung samples collected at 4 and 7 DPI, the sham vaccinated group yielded about 10^8^ and 10^5^ copies per gram tissues, respectively, while those receiving the 13.5 μg two-dose regimen were below the level of detection at both time points. The lung samples from those receiving the 1.5 and 4.5 μg two-dose regimens had a nearly 3 log reduction in viral sgRNA copies at 4 DPI and were below detection at 7 DPI. For the lung samples from those receiving the 1.5, 4.5 and 13.5 μg single-dose immunization, the viral loads at 4 DPI were not different from those of the sham vaccinated group while the loads at 7 DPI were below the threshold of detection. Notably, the lung samples from the 0.15 µg receiving one-dose or two-dose regimens had similar or even higher viral load as compared to those of the sham vaccinated group at either 4 or 7 DPI. However, the viral loads (sgRNA) at the nares were more diverse at 4 DPI among all groups, with one or two animals testing negative in most groups. The only group that achieved clearance of viral sgRNA in nasal samples at 7 DPI was the 13.5 µg two-dose immunization group. There were also some significant differences in the sgRNA medians between those receiving single doses of 4.5 and 13.5 µg or two doses of 0.15 µg immunization vs. sham group (Supplementary Table 10). These data demonstrated protection by MRT5500 immunization in this animal model, especially for prevention of lung infection and pathology. However, the immunization, even given at the high dose, could not completely prevent viral infection in the upper respiratory tract (nasal tissue). Vaccination may have an impact on transmission due to shortened duration and lower loads of viral shedding from the upper respiratory tract.

## Discussion

mRNA-based vaccine technology provides a rapid path for the evaluation of multiple antigen designs for vaccine candidates. Our focus encompassed evaluation of different design candidates encoding for various forms of the SARS-CoV-2 S protein with a goal to identify modifications that could be incorporated into a candidate for development. We hypothesized that neutralizing antibodies preferentially recognize conformational epitopes that can be displayed by structurally stable prefusion S antigen expressed on the cell surface. The prefusion structure could be further stabilized via removal of the furin cleavage site, which may prevent transition to a postfusion conformation^35^. We used ER localization, marked by KDEL staining, of S antigens in cells transfected with mRNA constructs and evaluation of trafficking to the cell surface to determine the impacts of prefusion stabilization mutations (2P and 6P), furin cleavage site mutations (GSAS), abrogation of ER retention signal (KLHYT)^45^ and various combinations of these mutations. Interestingly, the GSAS mutation appeared to have a dominant effect on ER localization: GSAS showed almost exclusive ER localization and minimal expression on the cell surface and in addition, it could overcome the abrogation mutation of a cytosol ER retention signal. Furthermore, GSAS in combination with 2P or 6P could reduce the effects of 2P and 6P on cell surface expression and promote ER localization. Coronavirus particles assemble at the ERGIC and bud into Golgi^60^, and furin could be active in the trans-golgi network, cell surface or endosome in processing viral glycoproteins during viral maturation^61^. Thus, the GSAS mutation observed in this study may indicate that furin cleavage likely was active within the ER, and that the cleavage could alter S antigen trafficking, allowing expression on the cell surface as shown with the 2P and 6P mRNA constructs.

To our surprise, however, the enhanced surface expression of prefusion stabilized S antigen was not associated with any benefit in immunogenicity in mice or NHPs. Although the 2P mRNA vaccine, similar to the recent mRNA vaccines approved for human use under Emergency Use Authorization, could elicit neutralizing titers approximated to the levels as previously reported in preclinical animal models^10,11,25,26,62^, the 6P vaccine utterly failed to elicit any detectable neutralizing titers in NHPs. On the contrary, increased ER localization as promoted by GSAS mutation appeared to be associated with enhanced immune responses as comparing to the WT or 2P constructs (Fig. 2). Furthermore, the GSAS mutation was able to partially negate the effect by the 6P mutation to restore the 6P/GSAS immunogenicity yielding low levels of neutralizing titers in NHPs. Lastly, the retention in the ER by the GSAS mutation appeared more dominant than the C-terminal ER signal (KLHYT) originally reported for SARS-CoV-1 virus^45^, as the abrogation of this ER signal did not show any impact on the ER staining of the 2P/GSAS/ALAYT mRNA construct. Although this mechanism of ER localization of GSAS mutants underlying the improved immunogenicity would require further investigation, the data supported selection of 2P/GSAS construct for further development for vaccine applications.

MRT5500, the construct with the 2P/GSAS mutations, demonstrated immunogenicity in NHPs at doses as low as 5 μg. MRT5500 also conferred protection in hamster model. There were two key points from this efficacy evaluation. First, the immunization, if used in a two-dose regimen at 1.5 μg or higher, could protect against the clinical COVID-19 disease. The protection was evident with a reduced and transient weight loss of <2% at 2–3 DPI, and no detectable lung pathology at 4 and 7 DPI. Other regimens including two doses of 0.15 μg two-dose and a single dose of 1.5 μg or higher, also showed certain levels of protection as compared to sham vaccinated control, but the weight loss in these hamsters were more noticeable and sustained through 7 DPI. In addition, these hamsters showed histopathological evidence of infection in their lungs at 4 and 7 DPI, albeit reduced when compared to sham control. Therefore, the effective regimen for prevention of symptomatic COVID-19 may need two vaccinations. Second, immunization although effective in preventing disease and pathology, was not effective in preventing viral infection, especially at the nasal tissues. While the hamsters, even with a single immunization of 1.5 μg or higher, could achieve viral clearance at 7 DPI, the nasal tissues from most hamsters were sampled positive for viral sgRNA at 4 and 7 DPI, indicative of active viral replication. These findings are similar to those previously reported for SARS-CoV infection in this model^55–58^. Protective efficacy of two vector-based vaccines Ad26 and YF17D were demonstrated against SARS-Cov-2 infection and severe clinical disease in hamsters^59,63^. Viral transmission post-immunization is a concern for all vaccines currently under development or authorized for emergency use.

The limitations of our study include the following points. First, we only assayed limited cytokines (Fig. 3c) in ELISPOT assays as contrary to the method of intracellular cytokine staining using flow cytometry. Although the cytokines IL-4, IL-5, or IL-13 could be used singularly or in combination as surrogate indicators of a T_H_2 response, it may not project a complete picture of the immune profile induced by MRT5500. Second, due to sample limitations, we could only measure neutralizing titers in hamsters prior to challenge, using our PsV neutralization assay. Thus, we could not analyze any immune parameters associated with the protection of histopathology changes or viral loads.

In summary, we have employed our mRNA technology for the rapid evaluation of vaccine candidates for COVID-19. Our results led to the selection of MRT5500 with 2P/GSAS modifications, distinct from the two mRNA vaccines recently approved for human use under emergency use authorization^4,5^. This candidate MRT5500 has been shown to be immunogenic by eliciting potent neutralizing antibodies in mice and NHPs, and T_H_1-biased cellular immune responses. In addition, the immunization is effective in a hamster model for the prevention of COVID-19. The candidate is positioned for further development in clinical studies.

## Methods

### mRNA synthesis, lipid nanoparticle formulation and expression assay

Messenger RNA incorporating coding sequences containing either the wild type (WT) sequence, stabilized pre-fusion mutant (2P)^64^, furin cleavage site mutant (GSAS)^38,65^, double mutant (2P, GSAS)), triple mutant (2P/GSAS/ALAYT), hexamutant (6P) and hexamutant with furin cleavage mutant (6P/GSAS) of the full length SARS-CoV-2 spike glycoprotein were synthesized by in vitro transcription employing RNA polymerase with a plasmid DNA template encoding the desired gene using unmodified nucleotides. The resulting purified precursor mRNA was reacted further via enzymatic addition of a 5′ cap structure (Cap 1) and a 3′ poly(A) tail of approximately 200 nucleotides in length as determined by gel electrophoresis. The vaccine sequence is based on Wuhan Hu-1 strain (Genbank accession MN908947). Preparation of mRNA/lipid nanoparticle (LNP) formulations was described previously^66^. Briefly, an ethanolic solution of a mixture of lipids (ionizable lipid, phosphatidylethanolamine, cholesterol and polyethylene glycol-lipid) at a fixed lipid and mRNA ratio were combined with an aqueous buffered solution of target mRNA at an acidic pH under controlled conditions to yield a suspension of uniform LNPs. Upon ultrafiltration and diafiltration into a suitable diluent system, the resulting nanoparticle suspensions were diluted to final concentration, filtered, and stored frozen at −80 °C until use. Expression of S-proteins from cells transfected with synthetic mRNAs was evaluated by Western blot. Briefly, 5×10^5^ HEK293 cells were transfected using 1 μg of mRNA complexed with Lipofectamine 2000 and allowed to incubate 20 h at 37 °C. Cells were harvested after incubation period, and lysates were analyzed by Western Blot as described elsewhere^67^.

### Visualization of S-proteins expressed in HeLa cells

Immunocytochemistry-immunofluorescence analysis of SARS Coronavirus S-protein was performed in HeLA cells transfected with mRNAs coding for either WT, 2P, GSAS, 2P/GSAS full-length SARS-CoV-2 S-proteins. Glass coverslips were placed in each well of a 12-well plate and coated with 100 μg/mL solution of poly-d-lysine to aid in cell adhesion. HeLa cells were seeded at a density of 2 × 10e5 cells per well and allowed to grow overnight to achieve ≥80% cell confluency prior to transfection. Mirus TransIT-mRNA Transfection Kit (Mirus, cat# MIR2225) was used to transfect cells with Covid-19 Spike Protein mRNAs. In brief, per one well 1 μg of mRNA was added to 100 μL of Opti-MEM I Reduced-Serum Medium followed by 2 μL of mRNA Boost Reagent and 2 μL of TransIT-mRNA reagent. The mixture was incubated at room temperature for 5 min for the complexes to form. These complexes were then added dropwise to different areas of each well containing HeLa cells in 1 mL complete growth medium. Cells were incubated with the complexes for 24 hours (h) to permit expression of mRNAs. After 24 h cells were fixed in 4% paraformaldehyde and subjected to antibody staining for the Spike Protein and KDEL, an endoplasmic reticulum (ER) marker^43,68^. After fixation cells were washed in phosphate buffer (PBS) followed by permeabilization in 0.5% Triton X-100 in PBS. Cells were then incubated for 0.3 h in a blocking solution consisting of 5% goat serum, 0.1% Triton X-100, 0.2% Bovine Serum Albumin, 50 mM ammonium chloride, 25 mM glycine, and 25 mM lysine in PBS. After washing with 0.1% Triton X-100 in PBS cells were incubated with primary antibodies against the Spike Protein (ThermoFisher, cat# MA5-35946) and KDEL (Abcam, cat# ab176333) for 1 h at room temperature. Cells were then washed 3× in PBS containing 0.1% Triton X-100 followed by incubation for 1 h at room temperature with secondary antibodies. Goat anti-Mouse IgG Alexa Fluor 488 (ThermoFisher cat# A32723) and Goat anti-Rabbit IgG Alexa Fluor 555 (ThermoFisher, cat# A32732) secondary antibodies were used at 4 μg/mL concentrations along with DAPI for visualization of nuclei. After incubation cells were washed 3 times in PBS containing 0.1% Triton X-100. Coverslips were then removed from wells and placed on glass slides with a drop of Aqua-Poly/Mount mounting medium (Polysciences, cat# 18606). After sealing the coverslips with nail polish, slides were imaged on a Carl Zeiss LSM880 confocal microscope equipped with a 40×1.2NA objective. Ten fields per slide were acquired, and images were analyzed in Visiopharm software to quantify colocalization of S-protein with ER marker.

### Non-human primate and mouse studies

Animal experiments were carried out in compliance with all pertinent US National Institutes of Health regulations and were conducted with approved animal protocols from the Institutional Animal Care and Use Committee (IACUC) at the research facilities.

The mouse studies were conducted at Covance Inc, Denver, PA. Female specific pathogen free BALB/c mice of 6–8-week-old were vaccinated in groups of 10, with 50 µL of the designated mRNA/LNP formulation into one hind leg for the prime (D0) and the contralateral hind leg for the boost (D21). Sera were collected on D-7, 14, 21, 28 and 35 from the orbital sinus or by exsanguination on D35 by the jugular vein/carotid artery. For cell-mediated response measurements, splenocytes from mice were collected on D35.

NHP studies were conducted at the University of Louisiana at Lafayette New Iberia Research Center. Cynomolgus macaques of Mauritian origin 2–6 years of age and weighing in a range of 2–6 kg were administered with 500 µL mRNA/LNP formulations via IM route into the deltoid of the right forelimb for the prime (D0) and the opposite forelimb for the boost (D21). Sera were collected on pre-immunization day (D-4), and post-immunization days 14, 21, 28, 35, 42 and, peripheral blood mononuclear cells (PBMCs) were isolated on D42. All immunizations and blood draws occurred under sedation with Ketamine HCl (10 mg/kg) or Telazol (4–8 mg/kg IM).

### Preparation of the SARS-CoV-2 and plaque reduction neutralization (PRNT) assay

SARS-Cov-2 USA-WA1/2020 isolate was passaged in Vero E6 cells from a seed stock (BEI Resources cat. no. NR-52281) at Bioqual. The challenge stock, which was assigned lot no. 061620-1000, was generated by the following method. The NR-52281 seed stock^69^ was diluted 1:200 in DMEM/2% FBS and added to Vero E6 cell (ATCC^®^ CRL-1586) monolayers (90–100% confluency) in T150 flasks. The cells were incubated with 5 mL diluted virus for 1 h at 37 °C, 5% CO_2_ with intermittent rocking. The virus was removed and replaced with DMEM, 2% FBS. The cells were incubated at 37 °C, 5% CO_2_ for 5 days when CPE was observed for 80–90% of the cells. The medium was collected and centrifuged at 1500 rpm for 10 min at 4 °C. The supernatant was maintained at 4 °C while collecting while collecting cells. Five (5) mL of DMEM, 2% FBS was added to each flask, the cells scraped cells off and collected into a conical 50 mL tube. The cells were spun down at 1500 rpm for 10 min at 4 °C and the resulting pellet resuspended in 5 mL of DMEM, 2% FBS. The cells were freeze-thawed twice to release virus and the resulting cell lysate combined with the supernatant. This was mixed well, 0.5 mL aliquots were prepared in cryovials and stored at ≤−70 °C.

PRNT assay with hamster sera samples was performed at Bioqual. After heat inactivation (30 min at 56 °C), hamster sera samples were serially diluted, mixed with SARS-CoV-2 viral stock and placed on 80–100% confluent monolayer of Vero E6 cells. Viral stock was tested to generate 30 plaque forming units (pfu) per well in 24-well plate of Vero E6 cells. Plates were incubated for 1 h at 37 °C in 5% CO_2_ incubator and media was discarded. Prewarmed 0.5% methylcellulose solution was added to each well and the plates were incubated at 37 °C in 5% CO_2_ for 72 h. Media was discarded and plates were washed once with Phosphate Buffered Saline (PBS). The plates were fixed with 400 μL ice cold methanol per well at −20 °C for 30 min. After fixation, the methanol was discarded, and the monolayers stained with 250 μL per well of 0.2% crystal violet (20% MeOH, 80% dH_2_O) for 30 min at ambient temperature. Plates were washed once with PBS and air dried for 15 min. The plaques in each well are recorded and the number of infectious units (pfu) calculated. The titers are reported as the serum dilutions that reduce the number of plaques by 50% (PRNT_50_).

### Hamster efficacy study

Eighty-eight (88) Female Syrian Golden Hamsters (hamsters), approximately 6–8 weeks of age and weighing in a range of 100–120 grams received either one or two doses of either of MRT5500 or sham control (n = 8 per group). Animals received either a single (on D0) or two (on D0 and D21) IM immunizations of MRT5500 at doses of 0.15, 1.5, 4.5 or 13.5 µg or sham (diluent) by the intramuscular route. On D49, all animals were challenged with 2.5 × 10^4^ plaque forming units (pfu), which was derived with one passage from USA-WA1/2020 (NR-52281, BEI Resources)^69^. Virus was administered as 100 μL by the intranasal route (50 μL in each nares). Body weights were assessed daily. All immunologic and virologic assays were performed blinded. On D4 and 7, a subset of animals was euthanized for tissue viral loads and pathology assays. Left lung samples were collected in 10% neutral buffered formalin and routinely processed for paraffin embedding. Paraffin blocks were sectioned at approximately 5 microns and stained using hematoxylin and eosin (H&E). H&E slides were blindly evaluated qualitatively and semi-quantitatively for pulmonary pathology (inflammatory infiltrates, pneumocyte and epithelial hyperplasia, mural and perivascular infiltrates obliterating normal lung parenchyma) by a board-certified veterinary pathologist. Lung sections were scored according to the percent area of tissue affected. A score of 0 was assigned to lungs that were consistent with normal tissue. A score of 1 was attributed to lung sections that revealed histopathology findings in <25% of the section. Similarly, if >25% but <50% of the parenchyma was involved, a score of 2 was assigned. A score of 3 was designated to those sections where more than 50% of the total section was affected.

Total RNA was extracted from tissue homogenates using RNA STAT-60TM (Tel-test “B”; Amsbio, Inc) in accordance with manufacturer instructions. SARS-CoV-2 E gene subgenomic mRNA (sgmRNA) was assessed by RT–PCR using primers SG-F (CGATCTTGTAGATCTGTTCCTCAAACGAAC) and SG-R (ATATTGCAGCAGTACGCACACACA) as well as probe FAM-ACACTAGCCATCCTTACTGCGCTTCG. Before RT–PCR, samples collected from challenged animals or standards (sham or naïve) were reverse transcribed using TaqMan RT-PCR kit (Bioline cat# BIO-78005). Amplification was performed in Applied Biosystems 7500 Sequence detector using the following program: 48 °C for 30 min, 95 °C for 10 min followed by 40 cycles of 95 °C for 15 s, and 1 min at 55 °C. A standard curve (the quantitative assay sensitivity was 50 copies) was used to calculate sgmRNA copies per gram tissue.

Hamster study was conducted in compliance with all relevant local, state, and federal regulations and were approved by the Bioqual Institutional Animal Care and Use Committee.

### Convalescent human sera

Convalescent serum panel (N = 93) was obtained from commercial vendors (Sanguine Biobank, iSpecimen and PPD). These subjects had a PCR positive diagnosis of COVID-19, and the serum samples were collected within 3 months following diagnosis.

### Enzyme-Linked Immunosorbent Assay (ELISA)

For NHP sera, Nunc MaxiSorb plates (Thermo Scientific, Cat#439454) were coated with custom made Geneart SARS-CoV S-GCN4 protein at 0.5 μg/mL in PBS overnight at 4 °C. For mouse and hamster sera, Nunc MaxiSorb plates were coated 2019-nCoV Spike protein (S1 + S2) ectodomain (Sino Biological, Cat# 40589-V08B1) was used as substrate and coated at 2 µg/mL concentration in bicarbonate buffer (CBB, Sigma, Cat# C3041-50CAP) overnight at 4 °C. Plates were washed 3–5 times with PBS-Tween 0.1% before blocking with 1% BSA in PBS-Tween 0.1% for 1 h at ambient temperature. Samples were plated at a 1:450 (for NHP), 1:20 (for hamster), or 1:50 (for mouse) initial dilution followed by 3–4 fold serial dilution in blocking buffer. Plates were washed 3–5 times after 1 h incubation at room temperature before adding 50 μL of 1:5000 Rabbit anti-human IgG (Jackson Immuno Research) for NHP sera, 50 μL of 1:10,000 Goat anti-Syrian Hamster IgG-heavy and light chain Antibody HRP Conjugated (Bethyl Cat # A140-101P) for hamster sera, or 100 μL of 1:2000 Goat anti-mouse H + L IgG (Abcam ab205719) for mouse sera to each well. Plates were incubated at room temperature for 1 h and washed 3–5×. Plates were developed using Pierce 1-Step Ultra TMB-ELISA Substrate Solution for 0.1 h and stopped by TMB stop solution (for NHP sera) or for ~10 min. using Sure Blue TMB 1-component (SERA CARE, KPL Cat# 5120-0077 or 5120-0075) and stopped by Stop solution (SERA CARE Sure Blue, KPL Cat# 5120–0024 or 5150-0020) (for mouse and hamster sera). Plates were read at 450 nm (for NHP and hamster) or 630 nm (for mouse) in SpectraMax or Thermo Labsystems Multiskan spectrophotometer plate readers. Antibody titers were reported as the highest dilution that is ≥0.2 Optical Density (OD) cutoff for NHP and hamster sera. For mouse sera, the endpoint antibody titer for each sample was determined as the highest dilution which gave OD value 3× higher than the background.

### Pseudovirus Neutralization Assay

Pseudoviruses containing Spike proteins from SARS-CoV-2 Wuhan Hu-1 strain, isolate WIV04 (Wuhan; Genbank accession MN996528.1) were prepared by Integral Molecular. Serum samples were diluted 1:4 in media (FluoroBrite phenol red free DMEM + 10% FBS + 10 mM HEPES + 1% PS + 1% Glutamax) and heat inactivated at 56 °C for 0.5 h. A further, 2-fold serial dilution of the heat inactivated serum were prepared and mixed with the reporter virus particle (RVP) -GFP (Integral Molecular) diluted to contain 300 infectious particles per well and incubated for 1 h at 37 °C. 96-well plates of 50% confluent 293T-hsACE2 clonal cells in 75 μL volume were inoculated with 50 μL of the serum/virus mixtures and incubated at 37 °C for 72 h. At the end of the incubation, plates were scanned on a high-content imager and individual GFP expressing cells were counted. The inhibitory dilution titer (ID_50_) was reported as the reciprocal of the dilution that reduced the number of virus plaques in the test by 50%. ID_50_ for each test sample was interpolated by calculating the slope and intercept using the last dilution with a plaque number below the 50% neutralization point and the first dilution with a plaque number above the 50% neutralization point. ID_50_ Titer = (50% neutralization point - intercept)/slope).

### Microneutralization assay

Serial two-fold dilutions of heat inactivated serum samples were incubated with a challenge dose targeting 100 50% tissue culture infectious dose (TCID_50_) of SARS-CoV-2 (strain USA-WA1/2020 [BEI Resources; catalog# NR-52281]) at 37 °C with 5% CO_2_ for 1 h (h). The serum-virus mixtures were inoculated into wells of a 96-well microplate with preformed Vero E6 (ATCC^®^ CRL-1586^TM^) cell monolayers and adsorbed at 37 °C with 5% CO_2_ for 0.5 h. Additional assay media was added to all wells without removing the existing inoculum and incubated at 37 °C with 5% CO_2_ for 2 days. After washing and fixation of the Vero E6 cell monolayers, SARS-CoV-2 antigen production in cells was detected by successive incubations with an anti-SARS-CoV nucleoprotein mouse monoclonal antibody (Sino Biological catalog# 40143-MM05), HRP IgG conjugate (Jackson ImmunoResearch Laboratories, catalog #115-035-062), and a chromogenic substrate. The resulting optical density (OD) was measured using a microplate reader. The reduction in SARS-CoV-2 infectivity, as compared to that in the virus control wells, constitutes a positive neutralization reaction indicating the presence of neutralizing antibodies in the serum sample. The 50% neutralization titer (MN ID_50_) was defined as the reciprocal of the serum dilution for which the virus infectivity was reduced by 50% relative to the virus control on each plate. The MN ID_50_ for each sample was interpolated by calculating the slope and intercept using the last dilution with an OD below the 50% neutralization point and the first dilution with an OD above the 50% neutralization point; MN ID_50_ Titer = (OD of 50% neutralization point−intercept)/slope.

### Cytokine ELISPOT analysis

For testing cytokine responses in mice, CTL ELISPOT kits (Mouse IFN-γ/IL-5 Double-Color enzymatic ELISPOT, Immunospot) were used according to the manufacture’s protocols. Briefly, freshly isolated splenocytes were resuspended in CTL-Test Media and incubated overnight at 300,000 cells per well with commercially available SARS-CoV-2 S peptide pools. PepMix™ SARS-CoV-2 (Spike Glycoprotein, Cat# PM-WCPV-S-1, JPT, Germany) peptide pool 1 and pool 2 were used at the final concentration of 2 µg/mL per well. Concanavalin A (CovA, Sigma C5275) at concentration of 1 µg/mL was used for a positive control stimulation. After overnight incubation, the plates were washed and developed per manufacturer instructions. Spots were scanned and analyzed by the CTL technical team. The number of cytokines producing cells per million cells was reported.

For testing cytokine responses in NHPs Monkey IFNɣ ELISPOT (CTL, cat# 3421M-4APW) and IL-13 ELISPOT kits (CTL, cat# 3470M-4APW) were used. Previously frozen PBMCs were washed, resuspended in culture medium provided by the kit, and enumerated. PepMix™ SARS-CoV-2 peptide pools as well as CovA were used for stimulation as described above. PBMCs were plated at 300,000 cells per well and stimulated overnight. After overnight incubation the plates were washed and developed per manufacturer instructions. The plates were dried overnight, scanned, and spots were counted using a CTL analyzer (Immunospot S6 Universal Analyzer, CTL). The data were reported as spot forming cells (SFC) per million PBMCs.

### Statistical analysis

Data were log-10 transformed prior to statistical analysis. All statistical tests were two-sided, and the nominal level of statistical significance was set to α = 5%. All analyses were performed on SEG SAS v9.4^®^. Statistical comparisons among different groups (different dose levels or constructs in a particular study) or between D35 and pre-bleed were conducted using mixed effect model for repeated measures, the model included group, day and their interactions, where day was specified as repeated measures.

When assessing pairwise correlations among IgG, MN, PsVNa in NHP study, we proposed a two-stage approach to separate the intra-and inter-variabilities for the repeated measures. Stage 1: we calculated the correlation coefficient for each individual subject based on observations over time per subject; Stage 2: we then estimated the mean and 95% CI of group correlation coefficient based on individual coefficient estimates. The analysis was based on log 10 transformed data.

Statistical comparisons among different groups (i.e. different dose levels) and the convalescent sera on D35 were conducted using either analysis of variance (ANOVA) or Wilcoxon Rank Sum Test.

Statistical comparisons in sgRNA copies in lung and nares between groups were conducted using Exact Wilcoxon signed-rank tests.

### Reporting summary

Further information on research design is available in the Nature Research Reporting Summary linked to this article.

## Supplementary information

Supplementary Information

Reporting Summary
