# Supplementary material for: Immunogenicity and efficacy of mRNA COVID-19 vaccine MRT5500 in preclinical animal models
Source: NPJ Vaccines. 2021 Apr 19;6:61. doi: 10.1038/s41541-021-00324-5 (PMC8055913; doi:10.1038/s41541-021-00324-5)
Supplement: Supplementary file 1 — Supplementary Information [file 41541_2021_324_MOESM1_ESM.pdf]

Supplementary Fig.1: Intracellular localization S antigen expressed in HeLa cells transformed with WT mRNA. Cells transformed with WT mRNA were fixed, permeabilized and stained with antibodies for Spike protein (green) and KDEL (red), an endoplasmic reticulum marker. Samples were then examined using confocal microscopy. The left column represents an overlay of Spike protein and KDEL, while the right column shows the spike protein signal superimposed on a transmitted light channel.

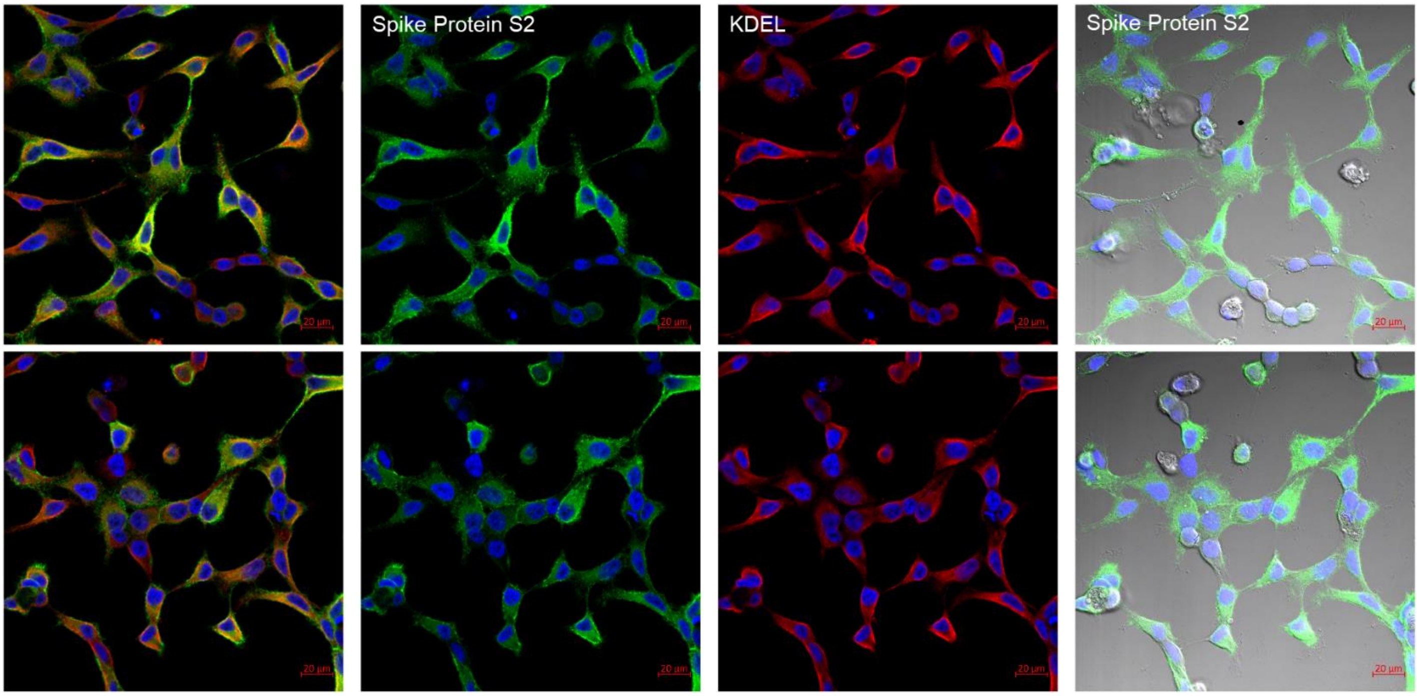

Supplementary Fig. 2: **Intracellular localization S antigen expressed in HeLa cells transformed with 2P mRNA.** Cells transformed with 2P mRNA were fixed, permeabilized and stained with antibodies for Spike protein (green) and KDEL (red), an endoplasmic reticulum marker. Samples were then examined using confocal microscopy. The left column represents an overlay of Spike protein and KDEL signals, while the right column shows the spike protein signal superimposed on a transmitted light channel.

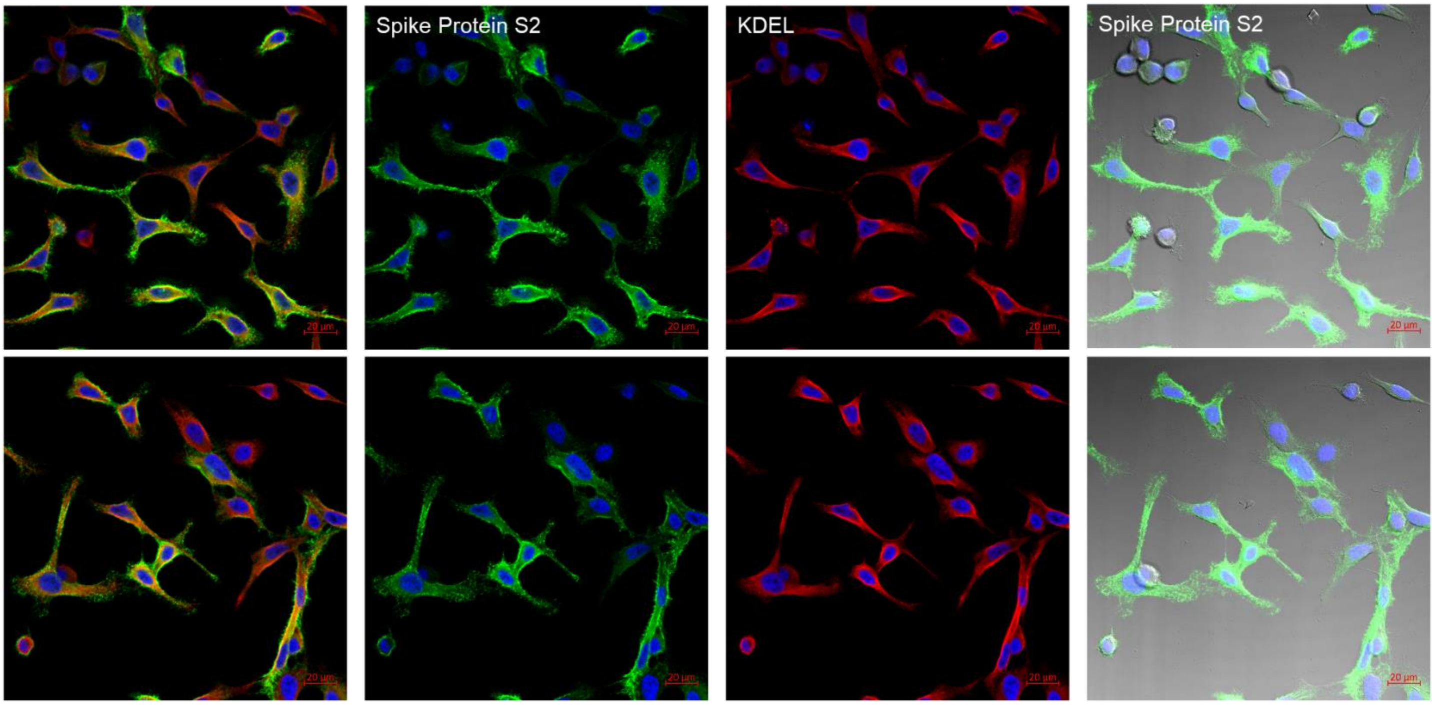

Supplementary Figure 3: Intracellular localization S antigen expressed in HeLa cells transformed with GSAS mRNA. Cells transformed with GSAS mRNA were fixed, permeabilized and stained with antibodies for Spike protein (green) and KDEL (red), an endoplasmic reticulum marker. Samples were then examined using confocal microscopy. The left column represents an overlay of Spike protein and KDEL signals, while the right column shows the spike protein signal superimposed on a transmitted light channel.

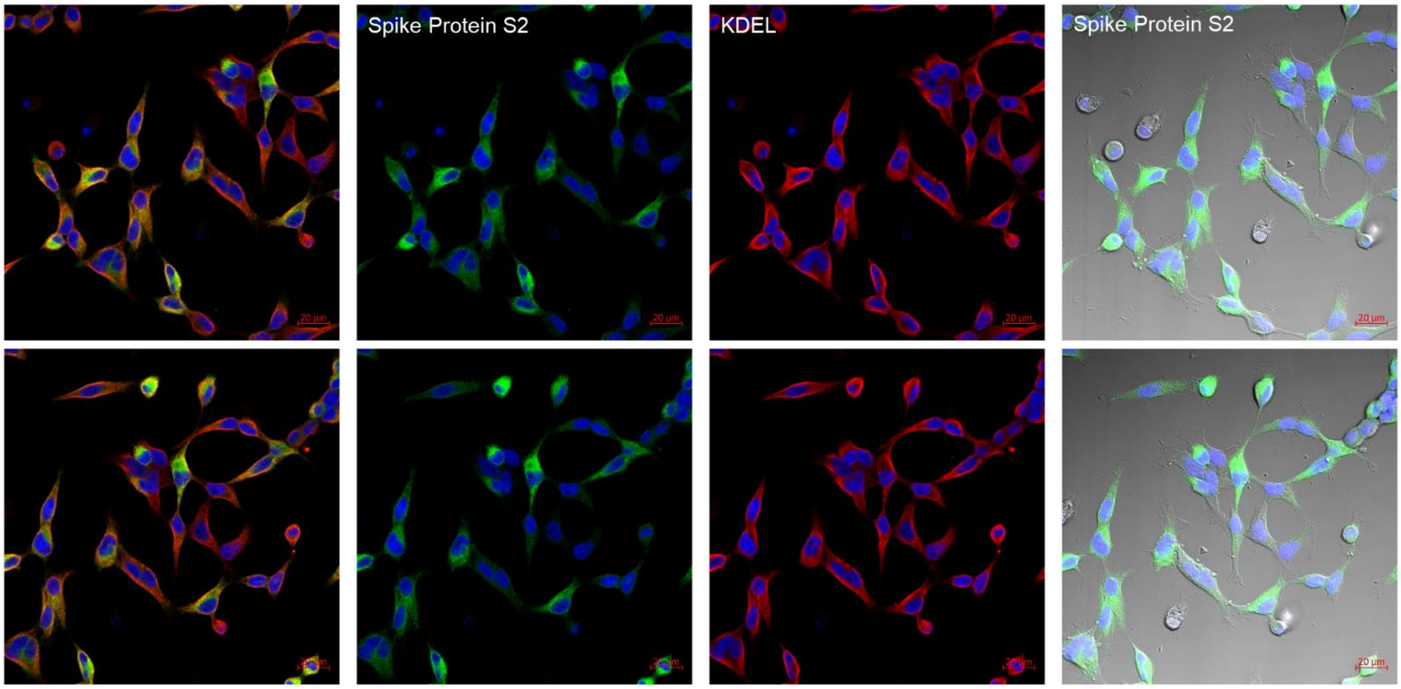

Supplementary Fig. 4: Intracellular localization S antigen expressed in HeLa cells transformed with 2P/GSAS mRNA. Cells transformed with 2P/GSAS mRNA were fixed, permeabilized and stained with antibodies for Spike protein (green) and KDEL (red), an endoplasmic reticulum marker. Samples were then examined using confocal microscopy. The left column represents an overlay of Spike protein and KDEL signals, while the right column shows the spike protein signal superimposed on a transmitted light channel.

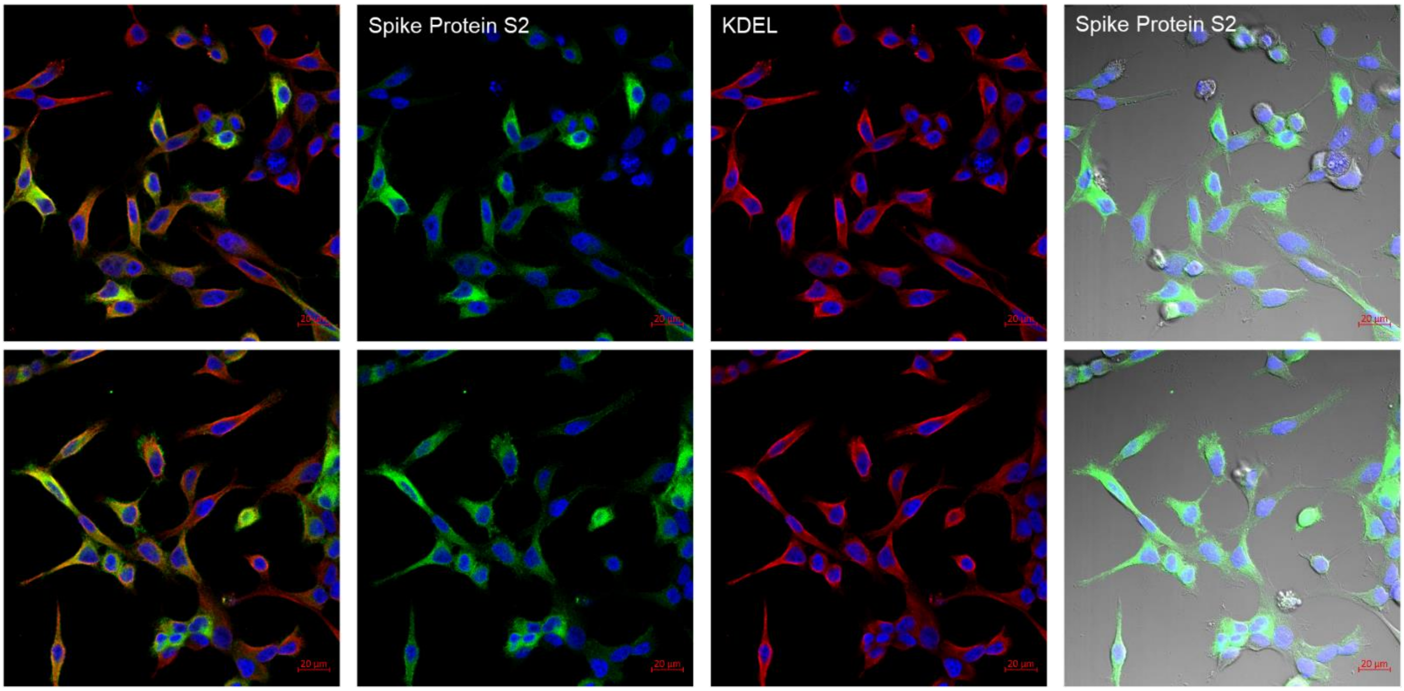

Supplementary Fig. 5: Intracellular localization S antigen expressed in HeLa cells transformed with 2P/GSAS/ALAYT mRNA. Cells transformed with 2P/GSAS/ALAYT mRNA were fixed, permeabilized and stained with antibodies for Spike protein (green) and KDEL (red), an endoplasmic reticulum marker. Samples were then examined using confocal microscopy. The left column represents an overlay of Spike protein and KDEL signals, while the right column shows the spike protein signal along superimposed on a transmitted light channel.

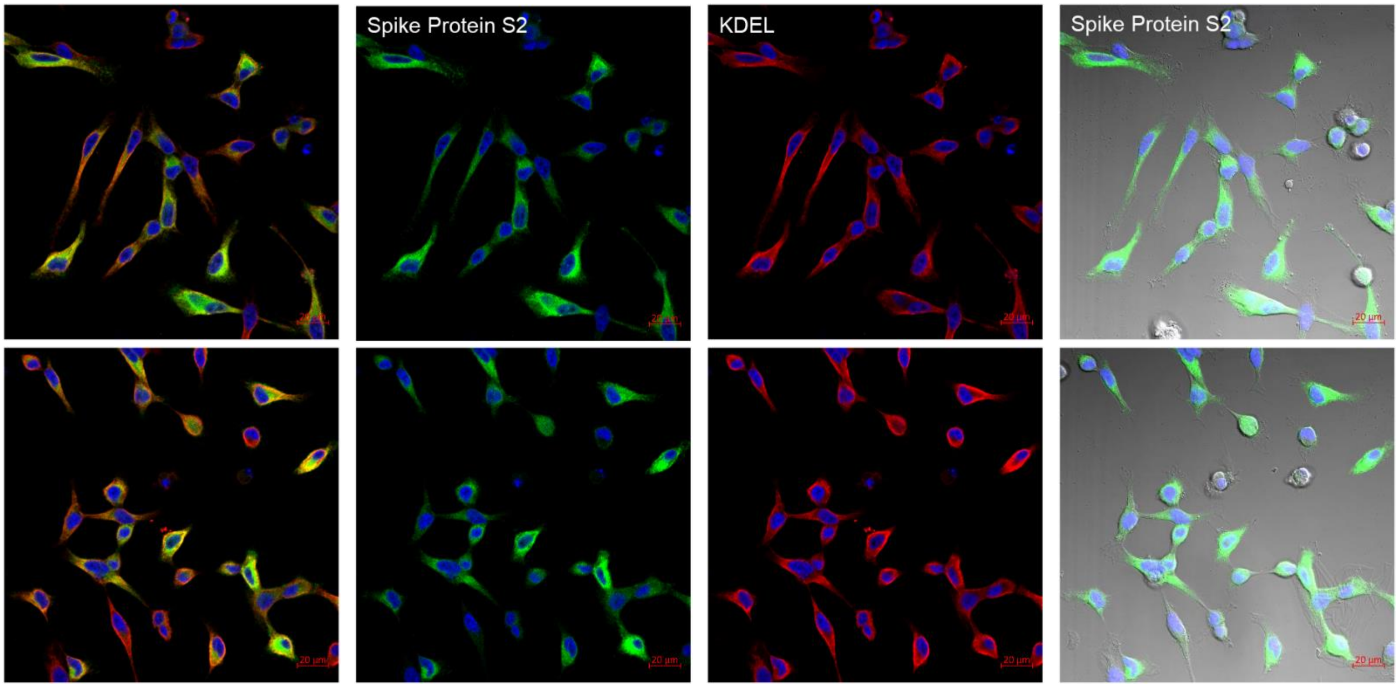

Supplementary Fig. 6: Intracellular localization S antigen expressed in HeLa cells transformed with 6P mRNA Cells transformed with 6P mRNA were fixed, permeabilized and stained with antibodies for Spike protein (green) and KDEL (red), an endoplasmic reticulum marker. Samples were then examined using confocal microscopy. The left column represents an overlay of Spike protein and KDEL signals, while the right column shows the spike protein signal superimposed on a transmitted light channel.

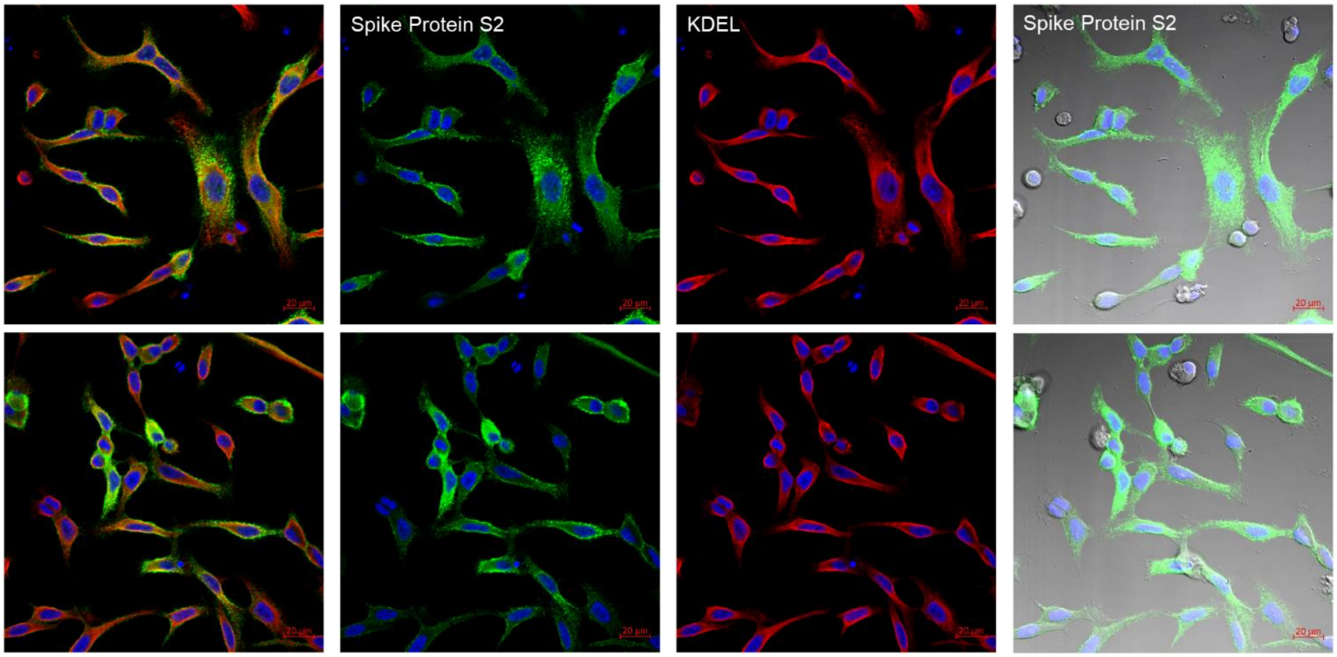

Supplementary Fig. 7: Intracellular localization S antigens expressed in HeLa cells transformed with 6P/GSAS mRNA. Cells transformed with 6P/GSAS mRNA were fixed, permeabilized and stained with antibodies for Spike protein (green) and KDEL (red), an endoplasmic reticulum marker. Samples were then examined using confocal microscopy. The left column represents an overlay of Spike protein and KDEL signals, while the right column shows the spike protein signal superimposed on a transmitted light channel.

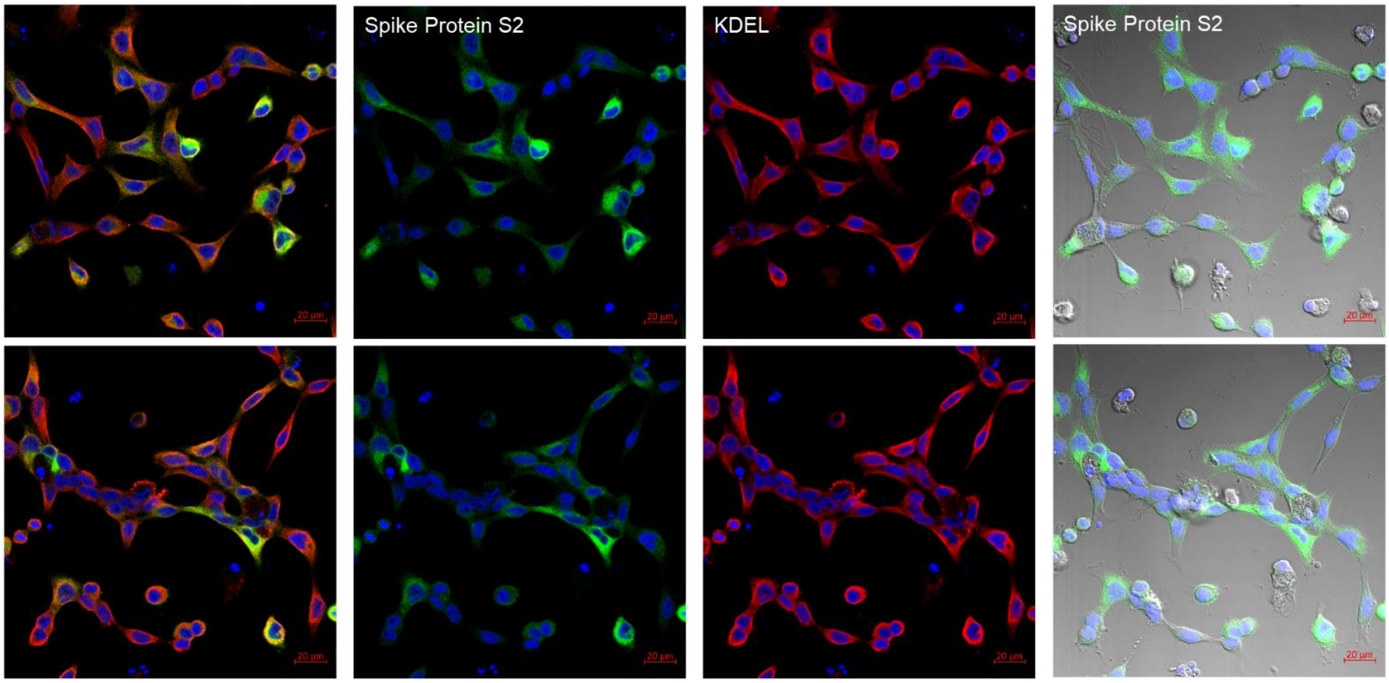

Supplementary Fig. 8: Relative degree (score) of surface localization of S-proteins based on visual inspection of HeLa cells transformed with mRNAs.

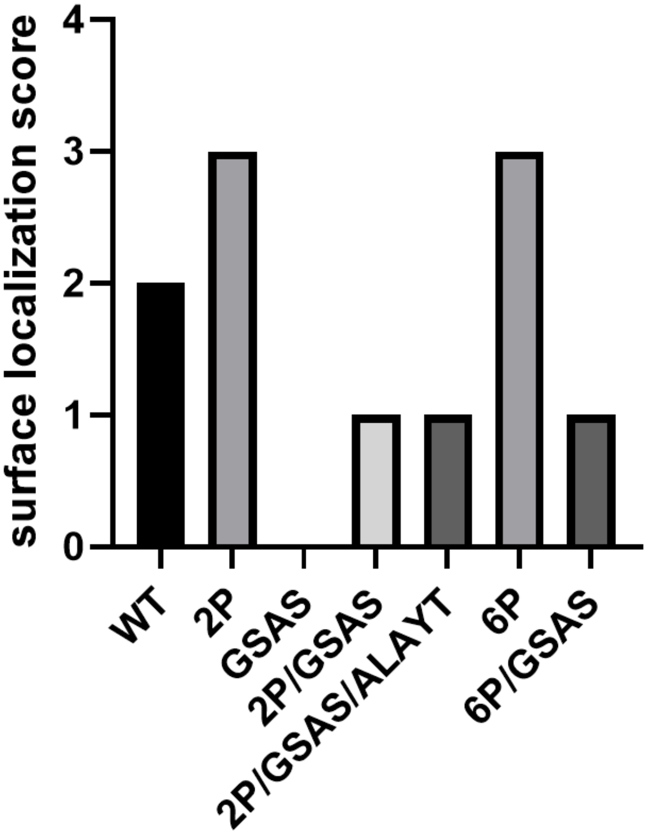

Supplementary Fig.9 Comparison of antibody binding responses against S antigen formulations in mice and NHPs. Serum binding antibody titers in mice (a) and NHPs (b) immunized with mRNA vaccines WT, 2P, GSAS, 2P/GSAS, 2P/GSAS/ALAYT, 6P, and 6P/GSAS. Briefly,BALB/c female mice (n=8) or Cynomolgus monkeys (n=4) were immunized twice three weeks apart with 0.4 µg or 5 µg of mRNA vaccines formulations respectively. 2P, GSAS and GSAS/2P (MRT5500) were tested in both animal models, while WT was tested only in mice.

Sera samples from pre-immunized animals (Pre-; D-4) as well as samples collected on D14, 21, 28, 35, 42 (NHP only) were tested for reactivity to recombinant S protein in ELISA or tested in a pseudoviurs neutralization assay (Fig. 2). Each dot represents an individual serum sample, and the line represents the geometric mean for the group. the dotted line below for each panel represents the lower limit of assay readout..

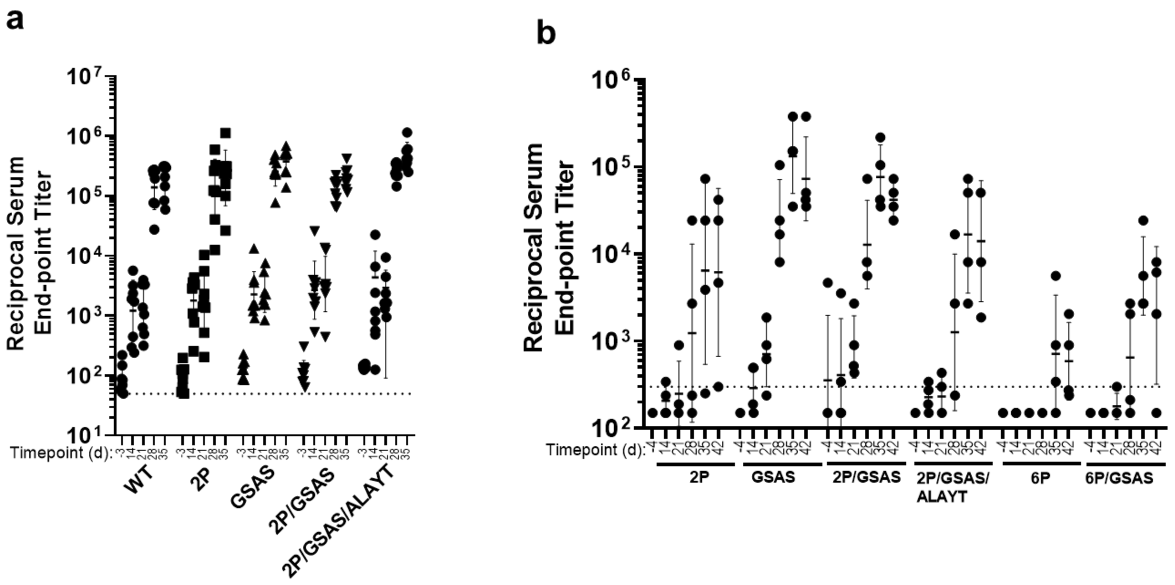

Supplementary Fig.10: MRT5500 induces T_H1-biased T-cell responses in mice. (**a**) IFNɣ and (**b**) IL-5 ELISPOT D35 data for the 5 and 10 µg doses. Following re-stimulation with S-protein peptides, pooled splenocytes MRT5500-immunized mouse groups secreted predominantly IFNɣ (T_H1) whereas IL-5 (T_H2) secretion was marginal.

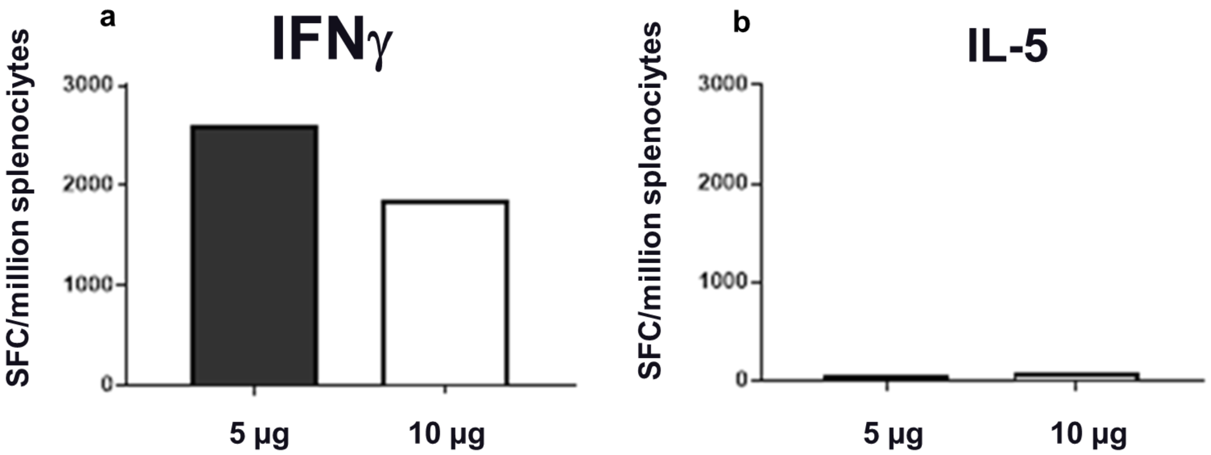

Supplementary Fig.11: MRT5500 elicited strong anti-spike antibody binding response in NHPs. Briefly, Nunc MaxiSorb plates were coated with SARS-CoV S-GCN4 protein (custom made at GeneArt) at 0.5 µg/mL in PBS overnight at 4°C. Plates were washed 3 times with PBS-Tween 0.1% before blocking with 1% BSA in PBS-Tween 0.1% for 1 h at ambient temperature. Samples were plated with 1:450 initial dilution followed by 3-fold, 7-point serial dilution in blocking buffer. Plates were washed 3 times after 1 h incubation at room temperature before adding 50 µL of 1:5000 Rabbit anti-human IgG (Jackson Immuno Research) to each well. Plates were incubated at room temperature for 1hr and washed 3x. Plates were developed using Pierce 1-Step Ultra TMB-ELISA Substrate Solution for 0.1 h and stopped by TMB stop solution. Plates were read at 450 nm in SpectraMax plate reader. Antibody titers were reported as the highest dilution that is ≥0.2 Optical Density (OD) cutoff.

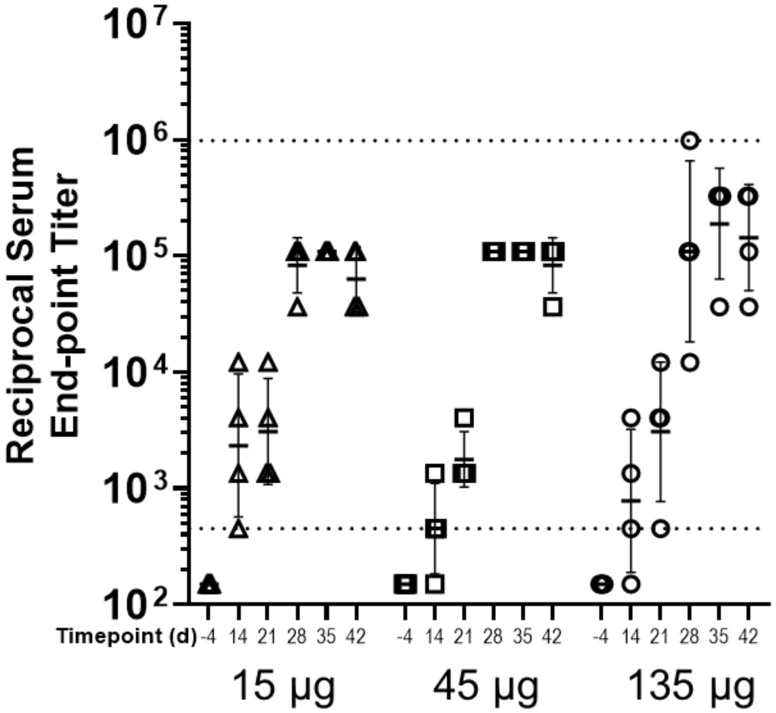

Supplementary Fig. 12: Strong correlations between individual NHP ELISA, PsV and MN time-point titers (see also Supplementary Table 2). Top panel A: 4 subjects in 15 µg dose; Middle panel B: 4 subjects in 45 µg dose; Bottom panel C: 4 subjects in 135 µg dose.

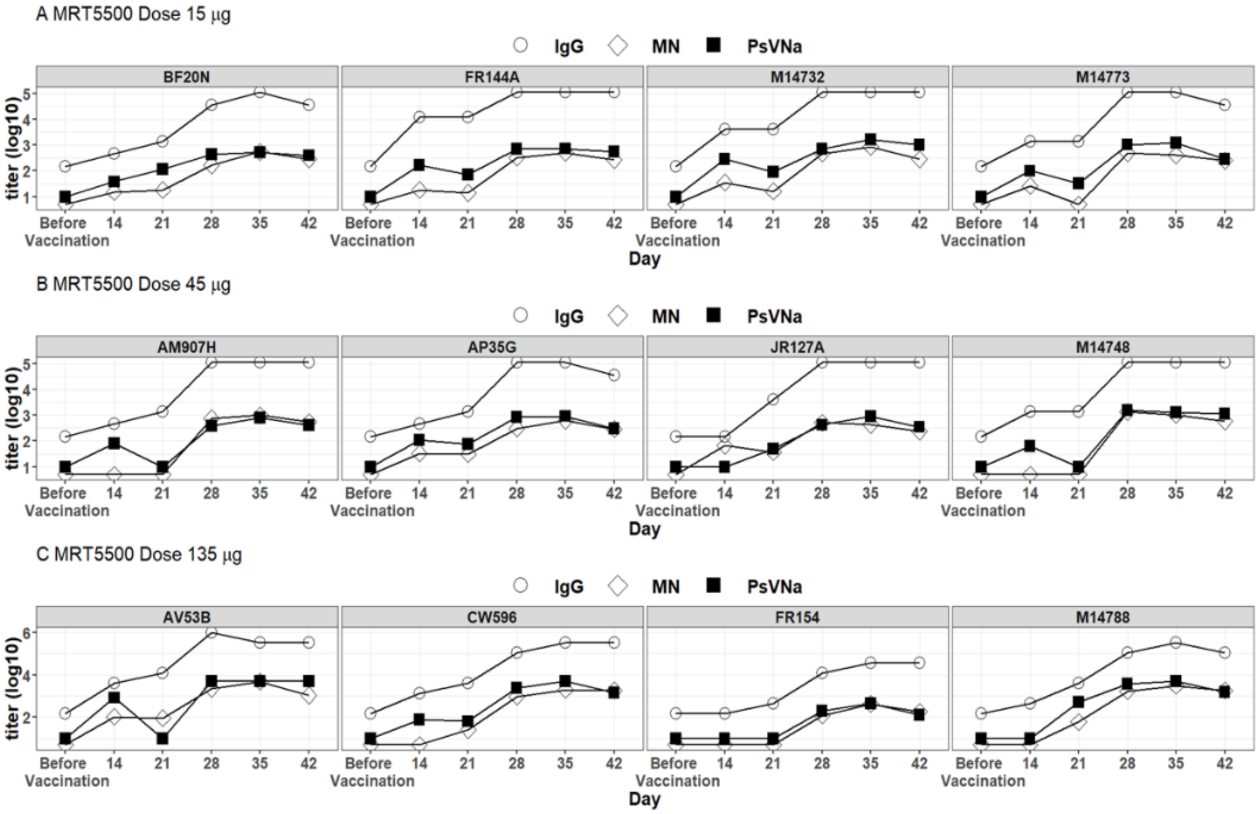

Supplementary Fig. 13: PsV titers in mice for the 1 µg, 5 µg and 10 µg dose levels of MRT5500 were significantly different from the Human Convalescent sera PsV titers

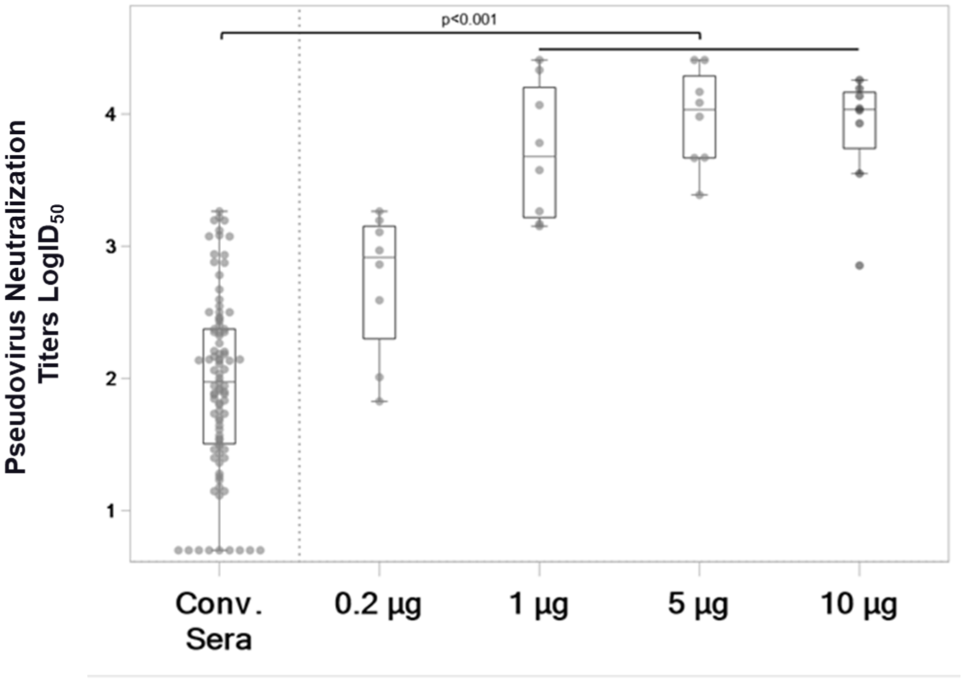

Supplementary Fig. 14: Comparison of D35 MRT5500 titers to human convalescent sera PsV titers in NHPs for the 15 µg, 45 µg and 135 µg dose levels of MRT5500 were significantly different from the Human Convalescent sera PsV titers.

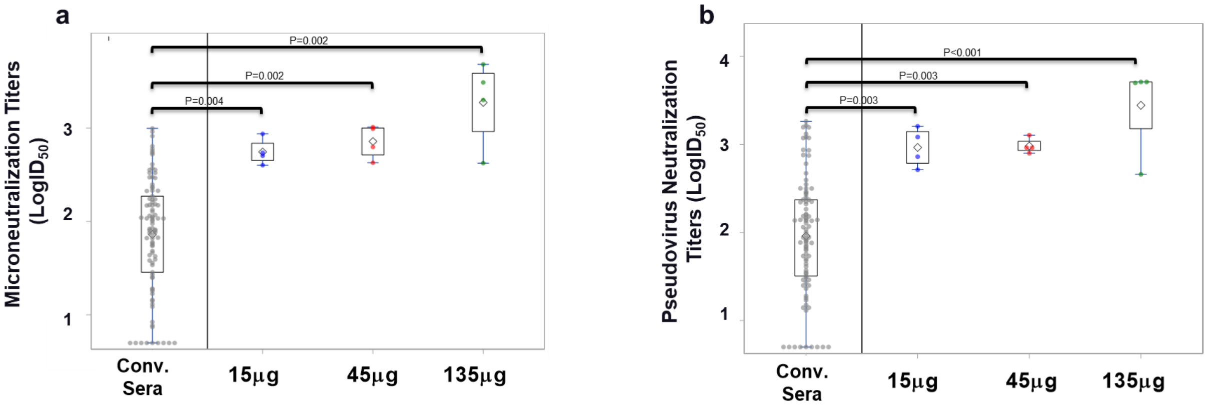

Supplementary Fig. 15: Protective efficacy of MRT5500 in hamster disease model. Low magnification representative photomicrographs (H&E) of lungs from hamsters receiving one or two doses of MRT5500 at increasing doses. In Sham (placebo) animals, more than 50% of the lung parenchyma is disrupted by marked inflammatory cell infiltrate, type II pneumocyte hyperplasia, multifocal hemorrhage, syncytial cells and cellular debris in hamsters treated once with the lowest dose represented by multifocal dark purple regions (arrows). Note, substantially reduced affected areas in lungs of hamsters receiving two doses and increasing doses. More than 50% of the lung parenchyma is disrupted in placebo treated mice as compared to naïve hamsters. Briefly, animals were immunized on D0, 21 with 0.15 µg, 1.5 µg, 4.5 µg, or 13.5 µg of MRT5500 and challenged on D42 with 10^6 pfu of SARS-CoV-2 intranasally (IN). Weight of

animals was monitored on daily basis.

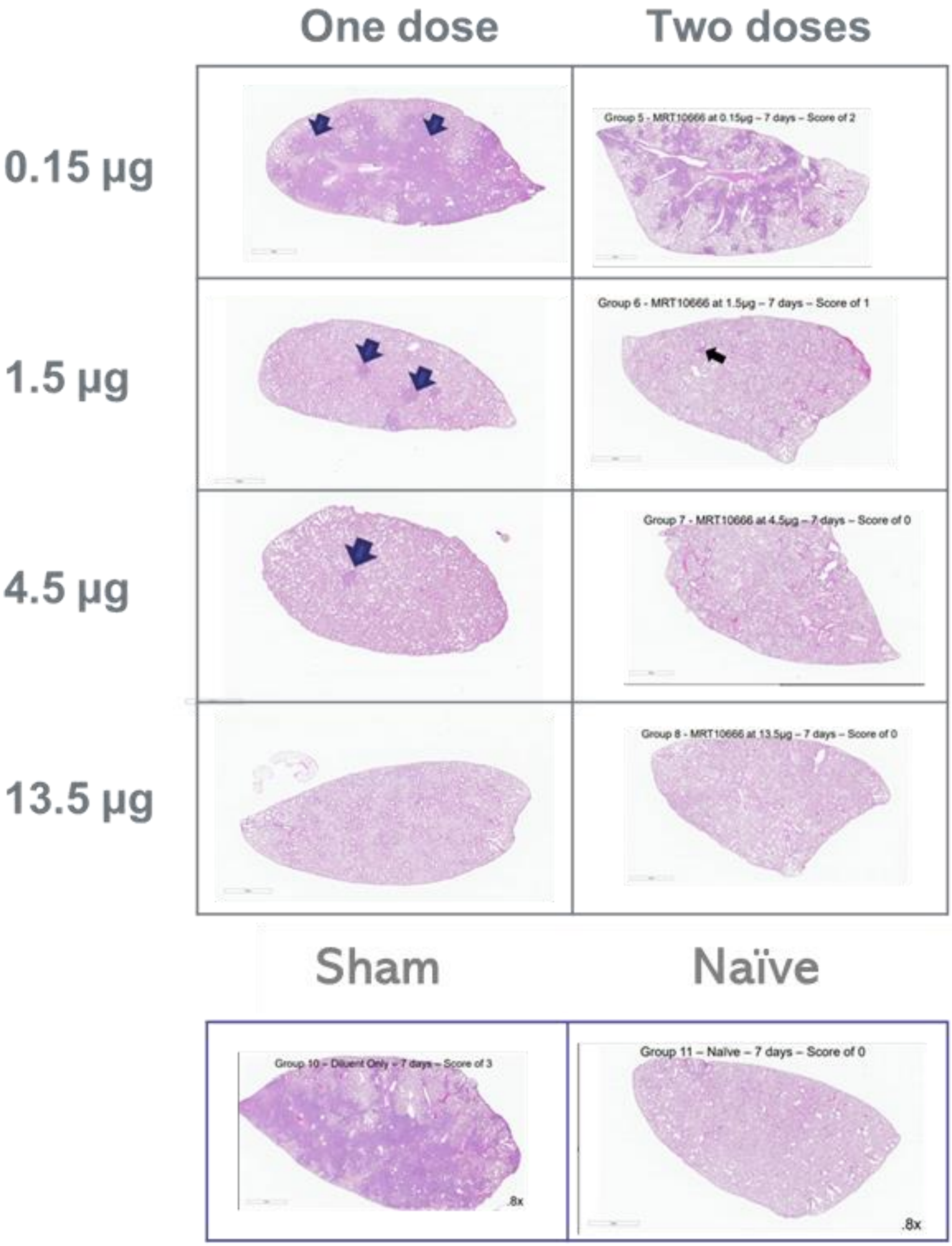

Supplementary Fig. 15 (see legend page 14)

Supplementary Fig.16: Pre-challenge MRT5500 neutralizing and ELISA titers in hamsters. Plaque reduction neutralization (PRNT50; **a**) and ELISA (**b**) titers in sera of MRT5500 vaccinated animals collected on D35 (one dose regimen) or D42 (two dose regimen). Briefly, hamsters were immunized either on D0 (one dose immunization) or D0, 21 (two doses immunizations) with 0.15 µg, 1.5 µg, 4.5 µg, or 13.5 µg of MRT5500 and challenged on D49 with 10^4 pfu of SARS-CoV-2 intranasally (IN).

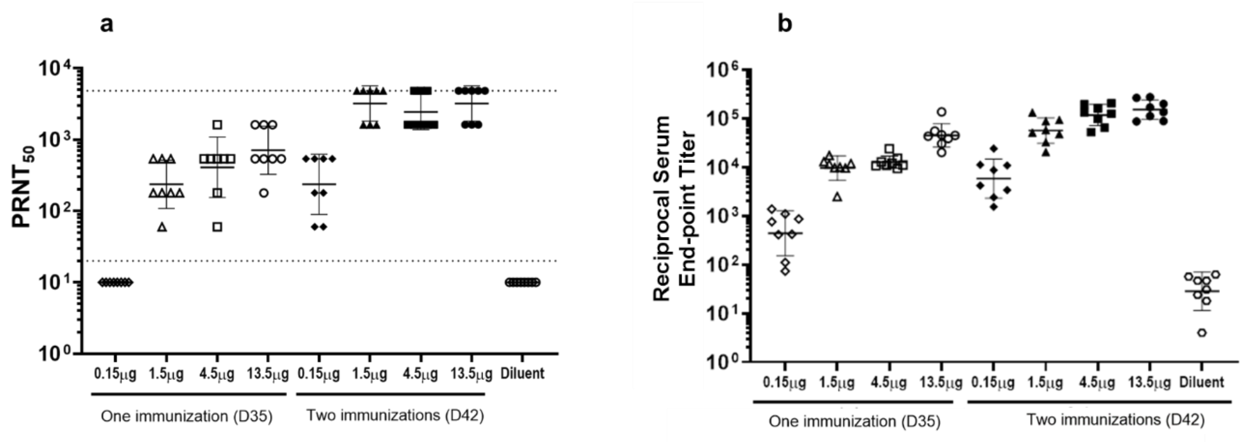

Supplementary Fig. 17: Example of D7 post challenge histopathology findings in lungs of hamsters. Representative photomicrographs (H&E) of lungs from a SARS-CoV-2 inoculated and placebo-treated hamster (a-c) and a naïve hamster (d-f). Normal lung parenchyma is disrupted by marked inflammatory cell infiltrate, type II pneumocyte hyperplasia, multifocal hemorrhage, syncytial cells, and cellular debris in infected hamster lung (b) as compared to normal parenchyma in naïve hamster lung (e). Bronchiolar epithelium (arrows) is markedly hyperplastic in infected lung sample (c) and histologically unremarkable in the naïve lung (f).

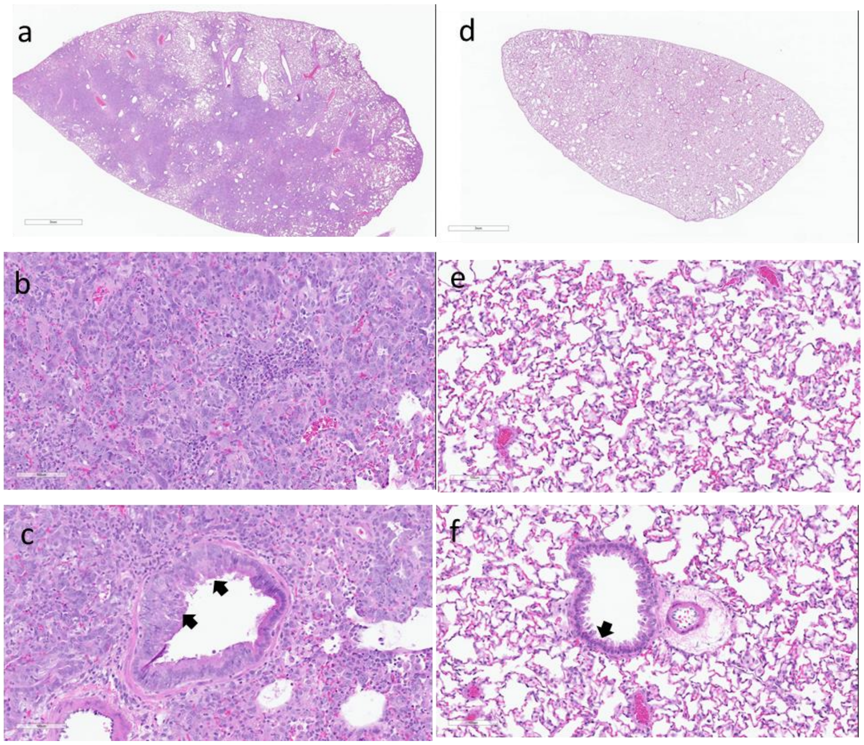

Supplementary Fig.18: Example of D7 post challenge histopathology in lungs of hamsters receiving one dose or two doses at 1.5 µg MRT5500. Representative lung photomicrographs (H&E). (**a**) Multiple foci of inflammatory cell infiltrate, type II pneumocyte hyperplasia, syncytial cells and cellular debris (arrows) are present in a hamster treated with a single 1.5 µg dose. (**c**) Higher magnification of boxed area in image a. Lung sections from a hamster treated with two 1.5 µg doses (b and d) appear histologically unremarkable.

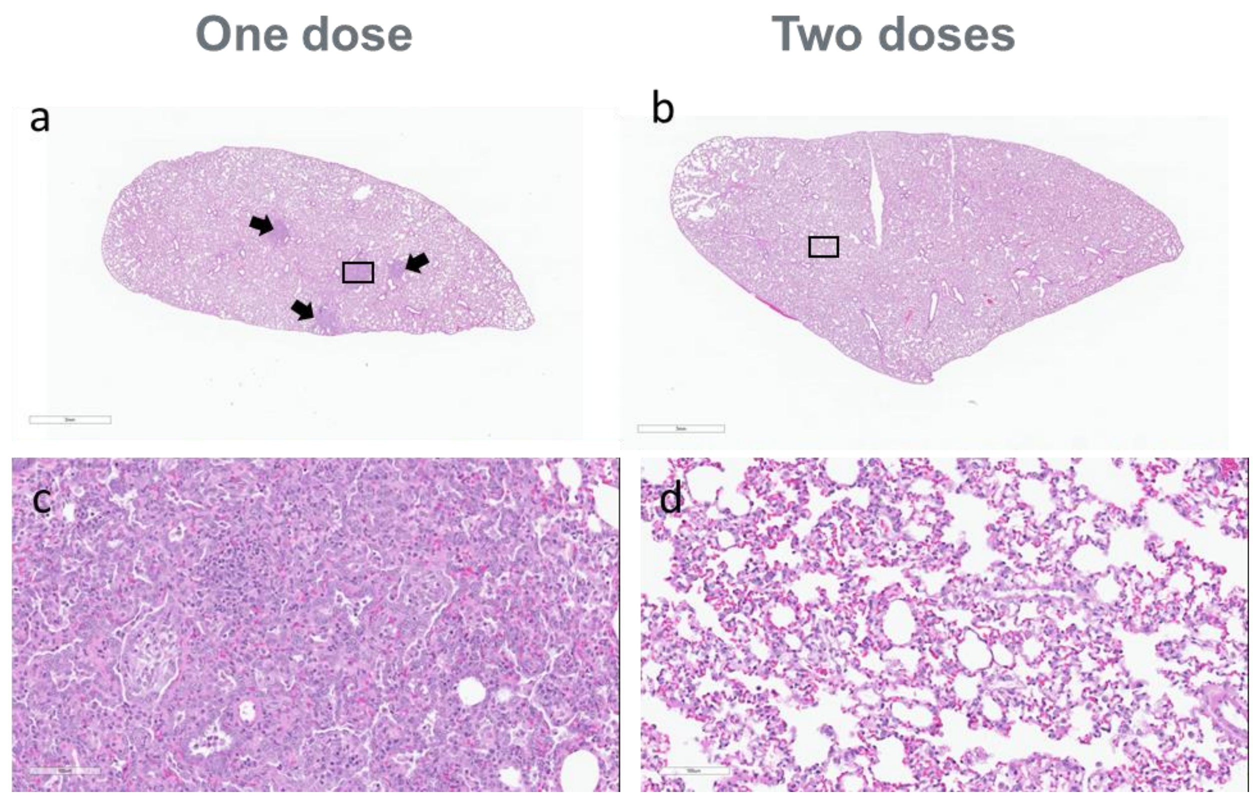

Supplementary Fig.19: Hamster challenge study. Individual weight loss by groups

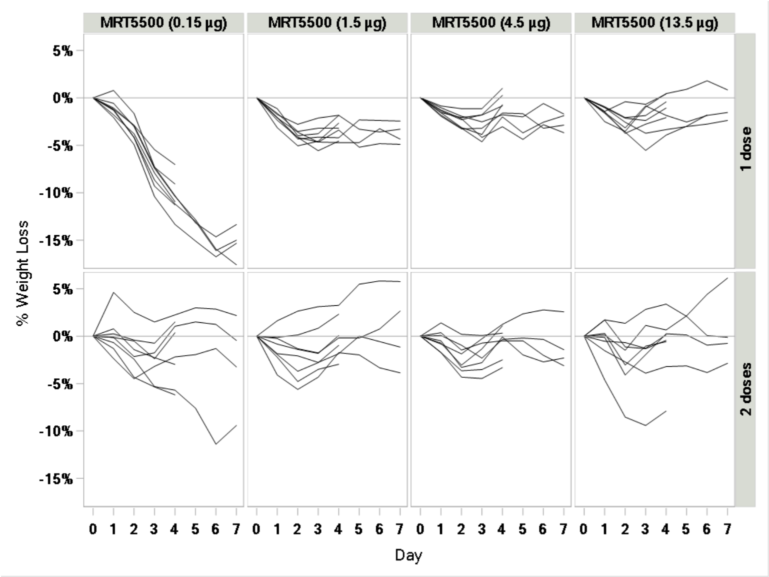

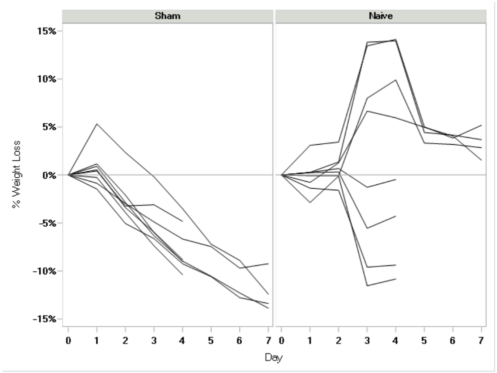

Supplementary Table 1: Pairwise dose comparison in PsV neutralization titers on D35 in mice. There were no statistically significant differences in PsV titers among the 1 µg, 5 µg and 10 µg dose levels, while at the lowest dose level (0.2 µg) PsV titers were significantly different from those obtained with the higher dose levels.

Pairwise dose comparisons PsVNa titers on D35 in mice	Fold Difference (95% CI)	p-value
0.2 µg vs. 1 µg	**/9.8 (4.4, 21.7)***	**p<0.0001**
0.2 µg vs. 5 µg	**/17.6 (7.9, 38.9)**	**p<0.0001**
0.2 µg vs. 10 µg	**/14.0 (6.3, 31.0)**	**p<0.0001**
1 µg vs. 5 µg	**/1.8 (0.8, 4.0)**	**p=0.1490**
1 µg vs. 10 µg	**/1.4 (0.6, 3.2)**	**p=0.3761**
5 µg vs. 10 µg	**x1.3 (0.6, 2.8)****	**p=0.5734**

Comparison: Group1 versus Group2
** /X.X: Group1 is X.X-fold lower than group2*
*** xX.X: Group1 is X.X-fold higher than group2*

Supplementary Table 2: Spearman Correlation Coefficients (SCC) between ELISA (IgG), Pseudoviral (PsV) and Microneutralization (MN) titers. SCC were conducted per individual animals (Suppl. Fig.4) and Means (95% CI) were calculated per dose (N=4) or all NHPs (N=12)

		Spearman Correlation Coefficient*		
Dose	N	ELISA and MN	ELISA and PsV	MN and PsV
15 µg	4	0.94 (0.90, 0.99)	0.95 (0.90, 1.00)	0.95 (0.90, 1.00)
45 µg	4	0.88 (0.82, 0.94)	0.90 (0.82, 0.97)	0.92 (0.81, 1.00)
135 µg	4	0.93 (0.83, 1.00)	0.88 (0.77, 0.98)	0.91 (0.84, 0.99)
Total	12	0.92 (0.89, 0.95)	0.91 (0.87, 0.94)	0.93 (0.90, 0.96)

*** Mean (95% CI)**

Supplementary Table 3: Hamster challenge data (1 dose regimen). Descriptive weight data analysis during 7 DPI

		1<br>Group			
		MRT5500 (0.15 µg)	MRT5500 (1.5 µg)	MRT5500 (4.5 µg)	MRT5500 (13.5 µg)
Weight D0 (g)					
	N	8	8	8	8
	NMiss	0	0	0	0
	Mean (SD)	142.8 (±18.6)	150.3 (±15.3)	149.7 (±10.5)	148.9 (±10.7)
	Min;Max	115.3;175.0	132.6;177.7	134.5;165.9	129.6;160.5
	Median [Q1;Q3]	141.9 [131.2;153.0]	146.6 [138.3;161.2]	150.7 [140.9;157.1]	151.3 [141.9;157.2]
Weight D1 (g)					
	N	8	8	8	8
	NMiss	0	0	0	0
	Mean (SD)	141.2 (±18.1)	147.2 (±14.7)	147.5 (±10.0)	146.7 (±10.5)
	Min;Max	116.2;172.9	129.8;173.5	133.1;163.3	127.6;157.9
	Median [Q1;Q3]	140.2 [129.0;151.2]	143.1 [136.3;157.8]	148.2 [139.4;154.4]	148.4 [140.2;155.5]
Weight D2 (g)					
	N	8	8	8	8
	NMiss	0	0	0	0
	Mean (SD)	137.9 (±18.2)	144.3 (±14.3)	146.1 (±9.5)	144.9 (±10.4)
	Min;Max	113.4;169.9	127.8;170.3	131.9;160.7	125.5;155.4
	Median [Q1;Q3]	137.1 [125.2;147.6]	140.3 [133.6;154.3]	146.5 [138.5;152.9]	148.4 [137.8;153.0]
Weight D3 (g)					
	N	8	8	8	8
	NMiss	0	0	0	0
	Mean (SD)	131.5 (±18.3)	144.1 (±13.6)	145.3 (±9.6)	145.3 (±9.3)
	Min;Max	106.9;162.2	126.4;167.8	131.1;159.5	128.5;155.0
	Median [Q1;Q3]	131.0 [118.3;142.3]	140.9 [134.2;154.1]	147.0 [137.1;152.0]	147.9 [138.6;152.9]
Weight D4 (g)					
	N	8	8	8	8
	NMiss	0	0	0	0
	Mean (SD)	128.1 (±18.5)	145.2 (±13.4)	148.1 (±10.7)	146.6 (±10.3)
	Min;Max	103.4;159.1	128.1;169.6	132.1;164.7	127.2;157.1
	Median [Q1;Q3]	126.8 [115.1;139.2]	142.3 [135.7;154.0]	149.7 [139.4;154.8]	149.0 [140.1;155.3]
Weight D5 (g)					
	N	4	4	4	4
	NMiss	4	4	4	4
	Mean (SD)	113.8 (±12.5)	142.1 (±15.6)	142.0 (±10.3)	147.4 (±14.3)
	Min;Max	100.1;127.5	125.7;155.5	131.8;153.8	126.3;157.0
	Median [Q1;Q3]	113.9 [103.5;124.2]	143.6 [128.7;155.5]	141.2 [133.5;150.5]	153.2 [138.5;156.4]
Weight D6 (g)					
	N	4	4	4	4
	NMiss	4	4	4	4
	Mean (SD)	110.7 (±11.1)	142.7 (±16.2)	142.9 (±9.7)	148.6 (±14.6)
	Min;Max	98.4;123.0	126.2;157.8	133.7;155.6	127.2;158.4
	Median [Q1;Q3]	110.7 [101.6;119.8]	143.4 [128.8;156.6]	141.2 [135.6;150.3]	154.3 [139.1;158.0]
Weight D7 (g)					
	N	4	4	4	3
	NMiss	4	4	4	5
	Mean (SD)	111.3 (±10.6)	142.3 (±15.6)	142.6 (±10.9)	145.4 (±15.6)
	Min;Max	99.9;124.6	126.1;156.0	132.2;156.7	127.6;156.9
	Median [Q1;Q3]	110.4 [103.3;119.4]	143.5 [128.9;155.7]	140.8 [134.2;151.1]	151.6 [127.6;156.9]

Supplementary Table 4. Hamster challenge data (2 dose regimen). Descriptive weight data analysis during 7 DPI

		2			
		Group			
		MRT5500 (0.15 µg)	MRT5500 (1.5 µg)	MRT5500 (4.5 µg)	MRT5500 (13.5 µg)
Weight D0 (g)					
	N	8	8	8	8
	NMiss	0	0	0	0
	Mean (SD)	146.2 (±17.0)	152.9 (±13.0)	149.0 (±14.3)	158.6 (±9.3)
	Min;Max	120.7;169.3	131.8;173.3	121.5;163.7	143.4;170.9
	Median [Q1;Q3]	144.2 [134.9;160.8]	152.1 [145.3;161.8]	151.4 [141.6;160.2]	158.8 [153.0;165.4]
Weight D1 (g)					
	N	8	8	8	8
	NMiss	0	0	0	0
	Mean (SD)	146.3 (±16.1)	151.1 (±13.5)	148.3 (±14.7)	158.0 (±10.8)
	Min;Max	121.0;165.9	126.4;170.1	120.9;163.9	141.2;171.4
	Median [Q1;Q3]	147.7 [134.4;159.7]	152.8 [143.4;160.0]	150.1 [139.8;160.9]	160.1 [148.9;166.9]
Weight D2 (g)					
	N	8	8	8	8
	NMiss	0	0	0	0
	Mean (SD)	143.6 (±14.9)	149.9 (±13.9)	145.6 (±14.8)	154.5 (±10.3)
	Min;Max	120.2;161.7	124.4;169.7	117.5;160.9	139.5;165.7
	Median [Q1;Q3]	144.6 [133.0;155.9]	153.8 [141.1;157.7]	147.9 [137.3;158.1]	154.7 [145.7;165.2]
Weight D3 (g)					
	N	8	8	8	8
	NMiss	0	0	0	0
	Mean (SD)	142.6 (±15.9)	150.4 (±13.3)	146.2 (±15.2)	155.7 (±12.5)
	Min;Max	119.8;163.9	126.1;168.5	118.1;162.1	137.8;169.9
	Median [Q1;Q3]	143.7 [129.8;154.9]	154.2 [141.8;158.6]	147.3 [138.0;159.4]	156.2 [144.6;168.2]
Weight D4 (g)					
	N	8	8	8	8
	NMiss	0	0	0	0
	Mean (SD)	143.9 (±15.8)	152.5 (±13.1)	148.2 (±14.8)	157.1 (±11.9)
	Min;Max	122.5;165.6	129.5;170.1	121.4;165.5	138.8;169.9
	Median [Q1;Q3]	144.6 [130.6;156.4]	155.5 [143.5;161.2]	149.2 [140.2;160.1]	158.7 [146.8;168.7]
Weight D5 (g)					
	N	4	4	4	4
	NMiss	4	4	4	4
	Mean (SD)	148.8 (±19.0)	154.2 (±18.4)	144.0 (±18.1)	158.9 (±14.3)
	Min;Max	121.7;166.0	129.2;173.3	119.1;162.5	138.9;171.6
	Median [Q1;Q3]	153.7 [136.7;160.9]	157.2 [142.6;165.9]	147.1 [132.8;155.2]	162.5 [148.9;168.9]
Weight D6 (g)					
	N	4	4	4	4
	NMiss	4	4	4	4
	Mean (SD)	147.7 (±21.7)	154.0 (±19.0)	143.2 (±17.7)	158.3 (±15.6)
	Min;Max	116.7;167.1	127.4;172.3	118.2;160.0	137.9;175.4
	Median [Q1;Q3]	153.4 [134.1;161.2]	158.2 [141.9;166.1]	147.3 [132.2;154.2]	160.0 [147.5;169.1]
Weight D7 (g)					
	N	4	4	4	4
	NMiss	4	4	4	4
	Mean (SD)	146.6 (±19.1)	154.3 (±19.4)	142.4 (±16.8)	159.4 (±16.1)
	Min;Max	119.3;163.8	126.7;171.3	118.7;158.2	139.3;178.3
	Median [Q1;Q3]	151.6 [134.9;158.3]	159.6 [141.5;167.1]	146.4 [131.7;153.2]	160.0 [148.4;170.4]

Supplementary Table 5. Hamster challenge data (Control groups). Descriptive weight data analysis during 7 DPI observation

		Sham	Naive
Weight D0 (g)			
	N	8	8
	NMiss	0	0
	Mean (SD)	149.2 (±14.3)	146.6 (±14.8)
	Min;Max	129.3;167.6	119.6;170.7
	Median [Q1;Q3]	152.7 [135.5;160.1]	147.1 [140.6;153.4]
Weight D1 (g)			
	N	8	8
	NMiss	0	0
	Mean (SD)	150.2 (±14.2)	146.2 (±13.0)
	Min;Max	130.0;168.3	123.3;165.8
	Median [Q1;Q3]	152.8 [136.9;162.0]	147.2 [139.1;153.8]
Weight D2 (g)			
	N	8	8
	NMiss	0	0
	Mean (SD)	145.2 (±13.9)	147.4 (±14.0)
	Min;Max	124.9;162.2	123.7;170.5
	Median [Q1;Q3]	148.4 [132.2;156.8]	148.0 [140.4;154.2]
Weight D3 (g)			
	N	8	8
	NMiss	0	0
	Mean (SD)	141.7 (±14.9)	148.3 (±13.0)
	Min;Max	121.6;162.4	125.2;167.0
	Median [Q1;Q3]	144.2 [127.2;153.5]	150.4 [141.1;155.7]
Weight D4 (g)			
	N	8	8
	NMiss	0	0
	Mean (SD)	137.8 (±14.9)	149.2 (±13.3)
	Min;Max	117.9;159.5	125.5;167.2
	Median [Q1;Q3]	140.5 [123.3;148.8]	151.5 [142.1;157.1]
Weight D5 (g)			
	N	4	4
	NMiss	4	4
	Mean (SD)	137.1 (±12.6)	145.2 (±13.2)
	Min;Max	118.6;146.9	125.6;153.2
	Median [Q1;Q3]	141.5 [129.4;144.9]	151.1 [137.7;152.8]
Weight D6 (g)			
	N	4	4
	NMiss	4	4
	Mean (SD)	134.1 (±12.2)	144.4 (±13.6)
	Min;Max	116.3;143.3	124.2;152.8
	Median [Q1;Q3]	138.5 [126.5;141.8]	150.3 [136.4;152.5]
Weight D7 (g)			
	N	4	4
	NMiss	4	4
	Mean (SD)	132.2 (±12.4)	143.6 (±12.3)
	Min;Max	114.2;142.3	125.8;152.1
	Median [Q1;Q3]	136.1 [124.5;139.9]	148.3 [135.4;151.9]

Supplementary Table 6. Hamster challenge data (1 dose regimen). % weight loss during 7 DPI observation

		1			
		Group			
		**MRT5500 (0.15 µg)**	**MRT5500 (1.5 µg)**	**MRT5500 (4.5 µg)**	**MRT5500 (13.5 µg)**
% Weight Loss (D1)					
	N	8	8	8	8
	NMiss	0	0	0	0
	Mean (SD)	-1.1 (±0.9)	-2.0 (±0.6)	-1.4 (±0.4)	-1.4 (±0.5)
	Min;Max	-2.1;0.8	-3.1;-1.1	-1.9;-0.9	-2.5;-0.9
	Median [Q1;Q3]	-1.2 [-1.6;-0.8]	-2.1 [-2.2;-1.7]	-1.5 [-1.7;-1.2]	-1.4 [-1.6;-1.0]
% Weight Loss (D2)					
	N	8	8	8	8
	NMiss	0	0	0	0
	Mean (SD)	-3.4 (±1.0)	-4.0 (±0.7)	-2.4 (±0.7)	-2.7 (±1.1)
	Min;Max	-4.9;-1.6	-5.1;-2.8	-3.3;-1.1	-3.7;-0.4
	Median [Q1;Q3]	-3.4 [-4.2;-2.9]	-4.1 [-4.3;-3.6]	-2.2 [-3.2;-2.0]	-2.9 [-3.6;-2.1]
% Weight Loss (D3)					
	N	8	8	8	8
	NMiss	0	0	0	0
	Mean (SD)	-8.0 (±1.5)	-4.1 (±1.1)	-2.9 (±1.3)	-2.4 (±1.7)
	Min;Max	-10.4;-5.5	-5.6;-2.1	-4.6;-1.1	-5.5;-0.7
	Median [Q1;Q3]	-7.7 [-9.0;-7.3]	-4.4 [-4.7;-3.5]	-2.9 [-4.0;-1.8]	-2.1 [-3.3;-0.9]
% Weight Loss (D4)					
	N	8	8	8	8
	NMiss	0	0	0	0
	Mean (SD)	-10.4 (±1.8)	-3.3 (±1.1)	-1.1 (±1.3)	-1.5 (±1.6)
	Min;Max	-13.3;-7.0	-4.7;-1.8	-3.0;1.0	-3.9;0.4
	Median [Q1;Q3]	-10.7 [-11.2;-9.7]	-3.3 [-4.4;-2.3]	-1.2 [-1.9;-0.2]	-1.5 [-2.7;-0.0]
% Weight Loss (D5)					
	N	4	4	4	4
	NMiss	4	4	4	4
	Mean (SD)	-13.5 (±1.0)	-3.9 (±1.3)	-2.9 (±1.3)	-1.9 (±1.9)
	Min;Max	-15.1;-12.8	-5.2;-2.3	-4.4;-1.7	-3.0;0.9
	Median [Q1;Q3]	-13.1 [-14.1;-12.9]	-4.0 [-5.0;-2.8]	-2.9 [-4.0;-1.8]	-2.8 [-3.0;-0.8]
% Weight Loss (D6)					
	N	4	4	4	4
	NMiss	4	4	4	4
	Mean (SD)	-15.9 (±0.9)	-3.5 (±1.0)	-2.3 (±1.2)	-1.2 (±2.0)
	Min;Max	-16.8;-14.7	-4.8;-2.4	-3.2;-0.6	-2.8;1.8
	Median [Q1;Q3]	-16.0 [-16.4;-15.3]	-3.4 [-4.2;-2.8]	-2.7 [-3.0;-1.6]	-1.8 [-2.3;-0.0]
% Weight Loss (D7)					
	N	4	4	4	3
	NMiss	4	4	4	5
	Mean (SD)	-15.3 (±1.7)	-3.8 (±1.1)	-2.5 (±0.9)	-1.0 (±1.7)
	Min;Max	-17.6;-13.4	-4.9;-2.4	-3.7;-1.7	-2.4;0.8
	Median [Q1;Q3]	-15.2 [-16.5;-14.2]	-3.8 [-4.6;-2.9]	-2.4 [-3.3;-1.8]	-1.5 [-2.4;0.8]

Supplementary Table 7. Hamster challenge data (2 dose regimen). % weight loss during 7 DPI observation

		2 Group			
		MRT5500 (0.15 µg)	MRT5500 (1.5 µg)	MRT5500 (4.5 µg)	MRT5500 (13.5 µg)
% Weight Loss (D1)					
	N	8	8	8	8
	NMiss	0	0	0	0
	Mean (SD)	0.1 (±2.1)	-1.2 (±1.7)	-0.5 (±1.1)	-0.4 (±2.0)
	Min;Max	-2.4;4.6	-4.1;1.6	-1.7;1.4	-4.6;1.7
	Median [Q1;Q3]	-0.1 [-1.0;0.5]	-1.3 [-2.1;-0.2]	-0.6 [-1.3;0.2]	-0.0 [-1.0;1.0]
% Weight Loss (D2)					
	N	8	8	8	8
	NMiss	0	0	0	0
	Mean (SD)	-1.7 (±2.3)	-2.0 (±2.7)	-2.3 (±1.5)	-2.5 (±2.9)
	Min;Max	-4.5;2.5	-5.6;2.6	-4.3;0.2	-8.5;1.4
	Median [Q1;Q3]	-1.8 [-3.4;-0.5]	-1.7 [-4.2;-0.6]	-2.5 [-3.5;-1.2]	-2.1 [-3.6;-0.9]
% Weight Loss (D3)					
	N	8	8	8	8
	NMiss	0	0	0	0
	Mean (SD)	-2.4 (±2.3)	-1.6 (±2.5)	-1.9 (±1.6)	-1.9 (±3.7)
	Min;Max	-5.4;1.5	-4.3;3.1	-4.5;0.1	-9.4;2.8
	Median [Q1;Q3]	-2.3 [-4.3;-1.3]	-2.3 [-3.1;-0.5]	-1.8 [-3.2;-0.5]	-1.3 [-2.9;0.0]
% Weight Loss (D4)					
	N	8	8	8	8
	NMiss	0	0	0	0
	Mean (SD)	-1.5 (±3.3)	-0.3 (±2.1)	-0.5 (±1.6)	-1.0 (±3.3)
	Min;Max	-6.2;2.2	-3.0;3.2	-3.3;1.2	-7.9;3.4
	Median [Q1;Q3]	-0.9 [-4.3;1.3]	-0.6 [-1.8;1.2]	-0.2 [-1.5;0.7]	-0.2 [-1.9;0.5]
% Weight Loss (D5)					
	N	4	4	4	4
	NMiss	4	4	4	4
	Mean (SD)	-1.3 (±4.7)	0.8 (±3.2)	-0.1 (±1.8)	0.3 (±2.5)
	Min;Max	-7.6;3.0	-2.0;5.5	-2.0;2.4	-3.1;2.1
	Median [Q1;Q3]	-0.2 [-4.8;2.2]	-0.1 [-1.1;2.7]	-0.3 [-1.2;1.1]	1.1 [-1.5;2.1]
% Weight Loss (D6)					
	N	4	4	4	4
	NMiss	4	4	4	4
	Mean (SD)	-2.1 (±6.4)	0.7 (±3.8)	-0.6 (±2.4)	-0.1 (±3.4)
	Min;Max	-11.4;2.9	-3.3;5.8	-2.7;2.8	-3.8;4.4
	Median [Q1;Q3]	-0.0 [-6.3;2.0]	0.1 [-2.0;3.3]	-1.2 [-2.4;1.2]	-0.4 [-2.4;2.2]
% Weight Loss (D7)					
	N	4	4	4	4
	NMiss	4	4	4	4
	Mean (SD)	-2.7 (±5.0)	0.8 (±4.2)	-1.1 (±2.5)	0.6 (±3.9)
	Min;Max	-9.4;2.2	-3.9;5.8	-3.1;2.6	-2.9;6.1
	Median [Q1;Q3]	-1.9 [-6.3;0.9]	0.7 [-2.5;4.2]	-1.9 [-2.7;0.6]	-0.4 [-1.8;3.0]

Supplementary Table 8. Hamster challenge data (Control groups). % weight loss during 7 DPI observation

		Sham	Naive
% Weight Loss (D1)			
	N	8	8
	NMiss	0	0
	Mean (SD)	0.7 (±2.1)	-0.1 (±1.7)
	Min;Max	-1.5;5.3	-2.9;3.1
	Median [Q1;Q3]	0.5 [-0.6;1.0]	0.1 [-1.1;0.3]
% Weight Loss (D2)			
	N	8	8
	NMiss	0	0
	Mean (SD)	-2.6 (±2.2)	0.7 (±1.5)
	Min;Max	-5.1;2.3	-1.6;3.4
	Median [Q1;Q3]	-3.1 [-3.7;-2.5]	0.5 [-0.1;1.3]
% Weight Loss (D3)			
	N	8	8
	NMiss	0	0
	Mean (SD)	-5.1 (±2.4)	1.7 (±10.1)
	Min;Max	-7.4;-0.2	-11.5;13.8
	Median [Q1;Q3]	-6.0 [-6.5;-4.0]	2.7 [-7.6;10.7]
% Weight Loss (D4)			
	N	8	8
	NMiss	0	0
	Mean (SD)	-7.7 (±2.4)	2.4 (±10.1)
	Min;Max	-10.4;-3.5	-10.8;14.1
	Median [Q1;Q3]	-8.9 [-9.1;-5.8]	2.7 [-6.8;11.9]
% Weight Loss (D5)			
	N	4	4
	NMiss	4	4
	Mean (SD)	-9.0 (±1.9)	4.4 (±0.8)
	Min;Max	-10.6;-7.2	3.3;5.0
	Median [Q1;Q3]	-9.0 [-10.6;-7.3]	4.7 [3.9;5.0]
% Weight Loss (D6)			
	N	4	4
	NMiss	4	4
	Mean (SD)	-10.9 (±1.9)	3.8 (±0.4)
	Min;Max	-12.8;-8.9	3.2;4.2
	Median [Q1;Q3]	-11.0 [-12.5;-9.3]	4.0 [3.5;4.1]
% Weight Loss (D7)			
	N	4	4
	NMiss	4	4
	Mean (SD)	-12.2 (±2.1)	3.3 (±1.5)
	Min;Max	-13.9;-9.2	1.5;5.2
	Median [Q1;Q3]	-12.9 [-13.6;-10.8]	3.3 [2.2;4.4]

Supplementary Table 9. Pairwise comparisons of PsVNa titers on D35 in NHPs (5 µg dose, see also Fig.2)

Group pairs	p-value (Wilcoxon Exact Rank Test)
**2P/GSAS vs 2P**	**0.3143****
**2P/GSAS vs GSAS**	**0.4857**
**2P/GSAS vs 2P/GSAS/ALAYT**	**0.4857**
**2P/GSAS vs 6P**	**0.0286**
**2P/GSAS vs 6P/GSAS**	**0.1143**
**2P/GSAS vs Convalescent Sera**	**0.0105***

* For comparison versus Convalescent Sera, Wilcoxon Rank Test was used (not the exact test)

** Two animals out of four in 2P group demonstrated PsVNa titers below lower limit of detection (see Fig.2)

Supplementary Table 10. sgmRNA copies in lungs and nares of MRT5500 vaccinated hamsters as compared to Sham (diluent) group (4 and 7 DPI)

4 DPI

Dose number	1 dose				2 doses			
	0.15 µg	1.5 µg	4.5 µg	13.5 µg	0.15 µg	1.5 µg	4.5 µg	13.5 µg
sgRNA copies in **Lungs**	> Sham p=0.0286	> Sham p=0.4857	= Sham p=1	< Sham p=0.0286	< Sham p=0.0571	< Sham p=0.0286	< Sham p=0.0286	< Sham p=0.0286
sgRNA copies in **Nares**	> Sham p=0.0571	< Sham p=0.8857	> Sham - p=0.1143	= Sham p=1	< Sham p=0.8857	< Sham p=0.4	< Sham p=0.4857	< Sham p=0.5429

7 DPI

Dose number	1 dose				2 doses			
	0.15 µg	1.5 µg	4.5 µg	13.5 µg	0.15 µg	1.5 µg	4.5 µg	13.5 µg
sgRNA copies in **Lungs**	> Sham p=0.1429	< Sham p=0.1429	< Sham - p=0.1429	< Sham p=0.1429	< Sham p=0.2571	< Sham p=0.1429	< Sham p=0.1429	< Sham p=0.1429
sgRNA copies in **Nares**	< Sham p=0.8857	< Sham - p=0.1143	< Sham - p=0.0571	< Sham p=0.0286	< Sham p=0.0286	< Sham p=0.3143	< Sham p=0.6	< Sham p=0.0286

- Tables represent the results of Wilcoxon Exact Test comparisons against Sham regarding sgRNA copies in Lung and Nares.
- ‘’=“ (equal), <’ (less then) or ‘>’ (more then) signs represent the directions of the difference as compared to Sham group
- DPI – days post infection (challenge) with SARS-CoV-2

Supplementary Table 11: List of acronyms

mRNA	messenger RNA
LNP	Lipid Nanoparticles
MRT5500	mRNA/LNP vaccine formulation
COVID-19	Coronavirus disease 2019
2019-nCoV	The 2019-novel coronavirus
SARS	Severe Acute Respiratory Syndrome
MERS-CoV	Coronavirus causing Middle East Respiratory Syndrome
HCoV-HKU1	Human coronavirus HKU1
SARS-CoV-2	Severe Acute Respiratory Syndrome Coronavirus 2, virus causing COVID-19
S-protein or S	Structural Spike glycoprotein of SARS-CoV-2
S-GCN4 protein	Recombinant S-protein containing a fusion of S-ectodomain with artificial trimerization domain GCN4 (custom made in GeneArt)
E-, M-, N-proteins	Proteins E, M, N of SARS-CoV-2
S1, S2	S1 and S2 subunits of S-protein
RBD	Receptor Binding Domain of S-protein
ACE2	Angiotensin-Converting Enzyme 2, cellular receptor of SARS CoV-1, 2
WT S-protein	Wild Type full length S-protein of SARS-CoV-2
2P S-protein	Pre-fusion stabilized mutant of full length S-protein of SARS-CoV-2
GSAS S-protein	Furin cleavage-site mutant of full length S-protein of SARS-CoV-2
2P/GSAS	S-protein double mutant containing 2P and GSAS
nAb	Neutralizing Antibodies
mAb	Monoclonal Antibodies
HEK293	Immortalized Human Embryonic Kidney 293 cells
HEK293T	The cell line, derived from the HEK293 cell line, that expresses a mutant version of the SV40 large T antigen
HeLa	Immortalized Human cervical cell line
kDa	kilo-Daltons, unit of protein mass

ER	Endoplasmic Reticulum
KDEL	Molecular marker of ER
ERGIC	Endoplasmic Reticulum–Golgi Intermediate Compartment
KLHYT	Intracellular ER retention signal of S—proteins of SARS-CoV-1, 2
BALB/c	Albino Laboratory-Bred/c strain of mice
ELISA	Enzyme-Linked Immunosorbent Assay
MN	Microneutralization
IgG	Immunoglobulin G
IgA	Immunoglobulin A
GMT	Geometric Mean Titers
GFP	Green Fluorescence Protein
RVP	GFP Reporter pseudoViral Particles
PsVNa	Pseudoviral neutralization assay
ID_{50}	Serum dilution providing 50% inhibition of RVP or WT SASR-CoV-2 entry
NHP	Non-Human Primate
PsV	Pseudovirus, pseudoviral
VAERD	Vaccine Associated Enhanced Respiratory Disease
RSV	Respiratory Syncytial Virus
RSV F protein	Major structural protein F of RSV
T_H1, T_H2	T-helper cells type 1 and 2
IFN-γ	Interferon gamma
IL-13	Interleukin 13
IL-5	Interleukin 5
rhIL-2	recombinant human Interleukin 2 (IL-2)
PBMC	Peripheral Blood Mononuclear Cell
ELISPOT	Enzyme-Linked Immune Absorbent Spot assay
RPMI 1640	The growth medium used in cell culture

Cap 1	Specially altered nucleotide on the 5′ end of some primary transcripts such as precursor messenger RNA
Poly(A) tail	A stretch of multiple adenosine monophosphates at 3′ end of some primary transcripts such as precursor messenger RNA
PBS	Phosphate Buffer
Triton X-100	Nonionic surfactant used for permeabilization of HeLa cells
IACUC	Institutional Animal Care and Use Committee
D	Day
IM	Intramuscular
$TCID_{50}$	50% Tissue Culture Infectious Dose
MN ID_{50} Titer	OD of 50% neutralization point - intercept)/slope
CovA	Concanavalin A
OD	Optical Density
CTL	Contact Laboratory Services
SFC	Spot Forming Cells
h	h
µg	Microgram
ng	Nanogram
µl	Microliter
mL	Milliliter
