# Supplementary material for: Immunogenicity and efficacy of mRNA COVID-19 vaccine MRT5500 in preclinical animal models
Source: NPJ Vaccines. 2021 Apr 19;6:61. doi: 10.1038/s41541-021-00324-5 (PMC8055913; doi:10.1038/s41541-021-00324-5)
Supplement: Supplementary file 2 — Reporting Summary [file 41541_2021_324_MOESM2_ESM.pdf]

nature research

Corresponding author(s): NPJVACINE-01220R

Last updated by author(s): Mar 11, 2021

Reporting Summary

Nature Research wishes to improve the reproducibility of the work that we publish. This form provides structure for consistency and transparency in reporting. For further information on Nature Research policies, see our Editorial Policies and the Editorial Policy Checklist.

Statistics

For all statistical analyses, confirm that the following items are present in the figure legend, table legend, main text, or Methods section.

n/a	Confirmed	
☐	☒	The exact sample size (*n*) for each experimental group/condition, given as a discrete number and unit of measurement
☐	☒	A statement on whether measurements were taken from distinct samples or whether the same sample was measured repeatedly
☐	☒	The statistical test(s) used AND whether they are one- or two-sided<br>*Only common tests should be described solely by name; describe more complex techniques in the Methods section.*
☐	☒	A description of all covariates tested
☐	☒	A description of any assumptions or corrections, such as tests of normality and adjustment for multiple comparisons
☐	☒	A full description of the statistical parameters including central tendency (e.g. means) or other basic estimates (e.g. regression coefficient) AND variation (e.g. standard deviation) or associated estimates of uncertainty (e.g. confidence intervals)
☐	☒	For null hypothesis testing, the test statistic (e.g. *F*, *t*, *r*) with confidence intervals, effect sizes, degrees of freedom and *P* value noted<br>*Give P values as exact values whenever suitable.*
☒	☐	For Bayesian analysis, information on the choice of priors and Markov chain Monte Carlo settings
☒	☐	For hierarchical and complex designs, identification of the appropriate level for tests and full reporting of outcomes
☐	☒	Estimates of effect sizes (e.g. Cohen's *d*, Pearson's *r*), indicating how they were calculated

Our web collection on statistics for biologists contains articles on many of the points above.

Software and code

Policy information about availability of computer code

Data collection	N/A
Data analysis	SAS version 9.4 was used for statistical analysis.

For manuscripts utilizing custom algorithms or software that are central to the research but not yet described in published literature, software must be made available to editors and reviewers. We strongly encourage code deposition in a community repository (e.g. GitHub). See the Nature Research guidelines for submitting code & software for further information.

Data

Policy information about availability of data

All manuscripts must include a data availability statement. This statement should provide the following information, where applicable:

- Accession codes, unique identifiers, or web links for publicly available datasets
- A list of figures that have associated raw data
- A description of any restrictions on data availability

All data from described experiments to be available from authors upon request

Field-specific reporting

Please select the one below that is the best fit for your research. If you are not sure, read the appropriate sections before making your selection.

☒ Life sciences ☐ Behavioural & social sciences ☐ Ecological, evolutionary & environmental sciences

For a reference copy of the document with all sections, see nature.com/documents/nr-reporting-summary-flat.pdf

Life sciences study design

All studies must disclose on these points even when the disclosure is negative.

Sample size	Due to ethical and technical reasons (limit of space; BL3 facility constrains, etc) all in vivo experiments were designed to minimize number of animals used in the studies. For rodent studies, N=8 for each group is generally considered sufficient for analysis. Housing is also generally limited to 4 animals per cage. For NHP studies, N=4 was chosen as a minimal group size due to cost and availability of NHPs. Statisticians were not consulted on the decision for sample size for these studies.
Data exclusions	No exclusion of data
Replication	We present data replicating studies across different animal models. Each study has multiple animals per group. And multiple timepoints were analyzed across various assays.
Randomization	For mice and hamsters, animals were assigned randomly. For NHP, animals were assigned to groups for an even distribution of ages, weights, and sexes and based on COVID-19 prescreen
Blinding	Investigators were not blinded during sample analysis as most samples were assayed at Sanofi Pasteur. Any testing conducted at CROs were made under a non-disclosure agreement.

Reporting for specific materials, systems and methods

We require information from authors about some types of materials, experimental systems and methods used in many studies. Here, indicate whether each material, system or method listed is relevant to your study. If you are not sure if a list item applies to your research, read the appropriate section before selecting a response.

Materials & experimental systems

n/a	Involved in the study	
☐	☒	Antibodies
☐	☒	Eukaryotic cell lines
☒	☐	Palaeontology and archaeology
☐	☒	Animals and other organisms
☒	☐	Human research participants
☒	☐	Clinical data
☒	☐	Dual use research of concern

Methods

n/a	Involved in the study	
☒	☐	ChIP-seq
☒	☐	Flow cytometry
☒	☐	MRI-based neuroimaging

Antibodies

Antibodies used	ThermoFisher, cat# MA5-35946<br>Abcam, cat# ab176333<br>ThermoFisher cat# A32723<br>ThermoFisher, cat# A32732<br>Bethyl Cat # A140-101P<br>Abcam ab205719<br>Sino Biological catalog# 40143-MM05<br>Jackson ImmunoResearch Laboratories, catalog #115-035-062
Validation	Validation was performed by vendors; recommendations. Antibodies were used in accordance to vendor recomendations.

Eukaryotic cell lines

Policy information about cell lines

Cell line source(s)	Vero E6 cell (ATCC® CRL-1586), HeLa ATCC® CCL-2™<br>ATCC® CCL-2™

Authentication	Authenticated by vendors.
Mycoplasma contamination	ATCC cell lines are guaranteed to be mycoplasma negative. We used standard antibiotic/antimycotic treatment in media as recommended by vendor.
Commonly misidentified lines (See ICLAC register)	N/A

Animals and other organisms

Policy information about studies involving animals; ARRIVE guidelines recommended for reporting animal research

Laboratory animals	Mice (Mus musculus), Balb/c, females, 8 weeks of age at study start, N=8 per group<br>Hamster (Mesocricetus auratus), Golden syrian, females, 8 weeks of age at study start, N=8 per group<br>NHP (macaca fascicularis), Mauritian cynomolgus macaques, male and female, 2-6 years of age at study start, N=4 per group
Wild animals	Study did not involve wild animals.
Field-collected samples	Study did not involve field collected non-human samples.<br>Convalescent serum panel (N=93) was obtained from commercial vendors (Sanguine Biobank, iSpecimen and PPD). These subjects had a PCR positive diagnosis of COVID-19, and the serum samples were collected within 3 months following diagnosis.
Ethics oversight	Animal experiments were carried out in compliance with all pertinent US National Institutes of Health regulations and were conducted with approved animal protocols from the Institutional Animal Care and Use Committee (IACUC) at the research facilities.

Note that full information on the approval of the study protocol must also be provided in the manuscript.
